# Applications of Modern Cell Therapies: The Latest Data in Ophthalmology

**DOI:** 10.3390/life15101610

**Published:** 2025-10-16

**Authors:** Ioannis Iliadis, Nadezhda A. Pechnikova, Malamati Poimenidou, Diamantis D. Almaliotis, Ioannis Tsinopoulos, Tamara V. Yaremenko, Alexey V. Yaremenko

**Affiliations:** 1 MSc in Ocular Surgery, School of Medicine, Aristotle University of Thessaloniki, 541 24 Thessaloniki, Greece; johniliadis96@gmail.com (I.I.);; 2 Department of Ophthalmology, General Hospital of Kilkis, 611 00 Kilkis, Greece; 3 Laboratory of Biomedical Engineering, School of Chemical Engineering, Aristotle University of Thessaloniki, 541 24 Thessaloniki, Greece; nikanobelevka@gmail.com; 4 Department of Biochemistry and Biotechnology, University of Thessaly, 382 21 Volos, Greece; 5 Saint Petersburg Pasteur Institute, 197101 Saint Petersburg, Russia; 6 School of Medicine, Aristotle University of Thessaloniki, 541 24 Thessaloniki, Greece; matina.poim@gmail.com; 7 Laboratory of Experimental Ophthalmology, School of Medicine, Aristotle University of Thessaloniki, 541 24 Thessaloniki, Greece; 8 Second Department of Ophthalmology, Papageorgiou General Hospital, School of Medicine, Aristotle University of Thessaloniki, 564 29 Thessaloniki, Greece; 9 Research and Clinical Center for Vision Restoration, 119021 Moscow, Russia

**Keywords:** ophthalmology, stem cell therapy, cell transplantation, clinical trials

## Abstract

Cell-based therapeutics are redefining interventions for vision loss by enabling tissue replacement, regeneration, and neuroprotection. This review surveys contemporary cellular strategies in ophthalmology through the lenses of therapeutic effectiveness, translational readiness, and governance. We profile principal sources—embryonic and induced pluripotent stem cells, mesenchymal stromal cells, retinal pigment epithelium, retinal progenitor and limbal stem cells—and enabling platforms including extracellular vesicles, encapsulated cell technology and biomaterial scaffolds. We synthesize clinical evidence across age-related macular degeneration, inherited retinal dystrophies, and corneal injury/limbal stem-cell deficiency, and highlight emerging applications for glaucoma and diabetic retinopathy. Delivery routes (subretinal, intravitreal, anterior segment) and graft formats (single cells, sheets/patches, organoids) are compared using standardized structural and functional endpoints. Persistent barriers include GMP-compliant derivation and release testing; differentiation fidelity, maturation, and potency; genomic stability and tumorigenicity risk; graft survival, synaptic integration, and immune rejection despite ocular immune privilege; the scarcity of validated biomarkers and harmonized outcome measures and ethical, regulatory, and health-economic constraints. Promising trajectories span off-the-shelf allogeneic products, patient-specific iPSC-derived grafts, organoid and 3D-bioprinted tissues, gene-plus-cell combinations, and cell-free extracellular-vesicle therapeutics. Overall, cell-based therapies remain investigational. With adequately powered trials, methodological harmonization, long-term surveillance, scalable xeno-free manufacturing, and equitable access frameworks, they may eventually become standards of care; at present, approvals are limited to specific products/indications and regions, and no cell therapy is the standard of care for retinal disease.

## 1. Introduction

Vision impairment is a major global health challenge, with tens of millions affected by blinding eye diseases. In 2020, an estimated 43.3 million people were blind worldwide, and another 295 million suffered moderate-to-severe visual impairment [1]. Age-related macular degeneration (AMD), glaucoma, corneal opacities, and diabetic retinopathy (DR) are among the leading causes of blindness, especially in aging populations. For example, AMD alone affects around 200 million people globally [2], a number expected to rise to 288 million by 2040 due to increasing life expectancy [2]. Likewise, glaucoma—the second leading cause of blindness worldwide—is projected to afflict over 111 million people by 2040, with more than 8 million already blinded in both eyes [3]. Corneal blindness is another critical issue: injuries and diseases of the cornea have left approximately 12.7 million people awaiting corneal transplants, yet donor tissue is severely scarce, with only one available cornea for every 70 needed globally [4]. Inherited retinal degenerations, such as retinitis pigmentosa (RP), although rarer, collectively impact roughly 1 in 3000 to 4000 people—about 2 million individuals worldwide [5]. These figures underscore the urgent need for new solutions in ophthalmology, as current standard treatments often fall short of restoring lost vision or keeping pace with the growing disease burden.

Conventional therapies in ophthalmology have achieved important successes but also face significant limitations. For instance, wet AMD can be managed with anti-VEGF drugs, but these require frequent invasive injections and primarily slow disease progression rather than restore vision. There is still no cure for advanced dry AMD (GA) aside from emerging experimental drugs [6]. In glaucoma, therapies like eye-drop medications or surgeries can lower intraocular pressure to protect the optic nerve, but any vision already lost from retinal ganglion cell death is permanent, as the central nervous system does not regenerate those cells [7]. Corneal transplantation can successfully restore sight for corneal opacities, but as noted, the severe shortage of donor corneas leaves the majority of patients untreated [4]. Furthermore, transplants carry risks of rejection and may fail over time [8,9]. Inherited retinal diseases (e.g., RP) and optic nerve injuries have historically had no effective treatments to replace dead photoreceptors or retinal ganglion cells (RGCs)—patients progressively lose vision despite supportive care. In summary, current therapies mostly manage symptoms or slow damage; they rarely regenerate or replace lost ocular cells. This creates a pressing need for regenerative strategies that can address the root cause of vision loss by restoring the cells and tissues that have been destroyed by disease.

Modern cell-based therapies—including, but not limited to, stem-cell approaches—represent a transformative approach to tackle the above challenges. Unlike traditional drugs or surgeries, cell-based therapies aim to replace or repair damaged cells in the eye, offering the possibility of true regeneration [10,11]. The history of cell therapies in ophthalmology began with pioneering attempts to transplant limbal stem cells (LSCs) to repair corneal damage in 1989 [12,13,14]. These initial interventions formed the basis for the further development of more sophisticated therapeutic approaches, which utilize various types of stem cells, such as embryonic stem cells (ESCs), induced pluripotent stem cells (iPSCs), mesenchymal stem cells (MSCs), and retinal progenitor cells (RPCs) [15]. The progress is reflected in the increasing number of preclinical and clinical studies aimed at evaluating the safety and therapeutic efficacy of these innovative approaches [15,16]. The advent of iPSC technology, for example, allows scientists to create patient-specific stem cells capable of differentiating into retinal or corneal cells, bypassing ethical issues of embryonic cells and reducing immune rejection risks [17,18]. Equally important, understanding of the eye’s immunology has improved—notably, many parts of the eye are immune privileged, meaning they can accept allogeneic (donor-derived) cells with a lower risk of rejection. This makes the eye an ideal target for off-the-shelf cell therapies that could be mass-produced. Modern cell therapies are significant today because they directly address the fundamental problem in blinding diseases: the loss of non-regenerating cells [17,18]. By injecting or transplanting new healthy cells (or tissues grown from them), it is possible to potentially restore functions that were previously irretrievable. This approach is poised to overcome the limitations of current treatments—instead of merely slowing degeneration, cell therapies could replenish retinal pigment epithelium, photoreceptors, corneal endothelium, or other critical cell types, thereby actually improving vision. The urgency is amplified by demographic trends (an aging global population at risk for AMD, glaucoma, etc.) and by the accumulating successes in preclinical and early clinical studies, which together have made regenerative ophthalmology one of the most promising frontiers in medicine today [17,19,20]. Modern cell therapies are being explored for multiple ocular conditions, especially those caused by the loss of specific cell populations. Key targets include AMD, glaucoma and optic neuropathies, corneal diseases and injuries, and inherited retinal degenerations.

In summary, modern cell therapies promise to fundamentally alter the course of diseases that were once deemed irreversible, turning back the clock on blindness at the cellular level.

## 2. Anatomy of the Eye and Its Physiology

### 2.1. Anatomy of the Eye

**Figure 1:**
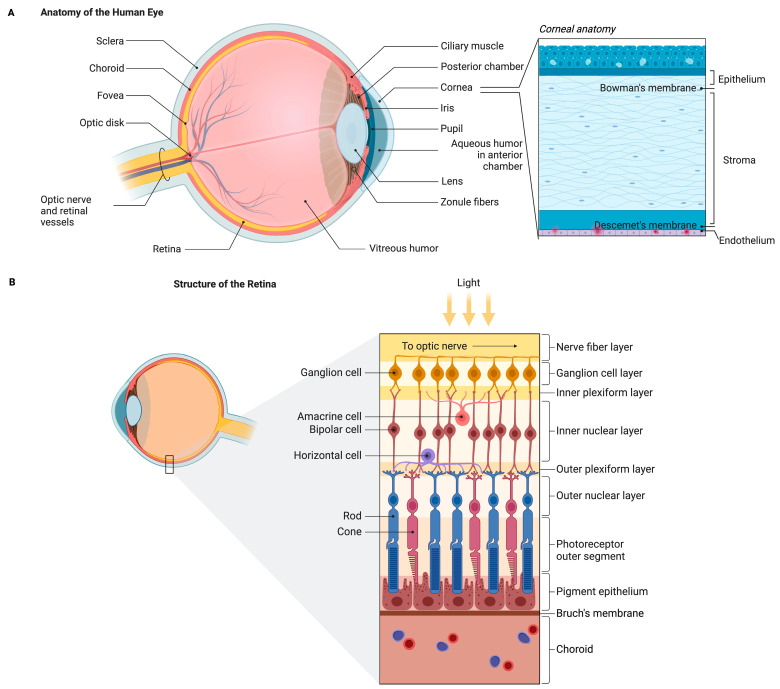
Organization of the human eye. (**A**) Anatomy of the human eye and corneal structure. (**B**) Cellular and layered structure of the retina. The layered organization of the retina depicts how light is processed through various cell types—photoreceptors (rods and cones), horizontal cells, bipolar cells, amacrine cells, and ganglion cells—culminating in signal transmission to the optic nerve. The retinal layers, such as the nerve fiber layer, nuclear layers, and plexiform layers, are also labeled (created by https://www.biorender.com/, accessed on 5 September 2025).

The human eye, as a highly specialized sensory organ, has a complex anatomical and functional organization that enables it to perceive light and process visual information [21]. The structure of the eye includes the following: the cornea, sclera, iris, pupil, lens, retina, optic nerve, and several fluid-filled chambers (Figure 1A) [21,22,23,24]. The cornea is a transparent, vascular tissue that serves as the main refractive surface and optical interface of the eye. It consists of three cell layers: epithelium, stroma, and endothelium separated by two non-cellular membranes, Bowman’s layer and Descemet’s membrane. The corneal epithelium provides a protective barrier against external attacks, while the stroma, which comprises approximately 90% of the thickness of the cornea, maintains structural integrity and transparency [15]. The endothelium regulates the hydration of the layer through active ion transport mechanisms. The density of endothelial cells averages around 3000 cells/mm^2^ in adults, gradually decreasing from approximately 4000 cells/mm^2^ at birth to 2000 cells/mm^2^ in elderly individuals [25]. More deeply, the iris regulates the amount of incoming light, while the lens focuses light stimuli on the retina. The retina, located at the back of the eyeball, is a multilayered neural tissue that houses the photoreceptors (cones and rods) responsible for converting light into electrical signals (Figure 1B). These signals are processed through successive layers of intermediate nerve cells (bipolar, amacrine) and are ultimately transmitted to the RGCs, whose axons form the optic nerve. Müller cells, a special type of glial cell, play an essential role in the structural and functional support of nerve cells. In addition, the space between the lens and the retina is filled with the vitreous body, a transparent, gel-like substance that helps maintain the shape and clarity of the eye [23,26].

### 2.2. Brief Physiology of the Eye

Vision begins when the cornea and lens function as a dynamic optical system that focuses light onto the retina. Pupil size is governed by midbrain parasympathetic circuits (Edinger–Westphal nucleus) that mediate the light–near response, and accommodation depends on parasympathetic activation of the ciliary muscle [23,24]. In the retina, photoreceptors convert photons into graded signals via a cascade of visual pigments. These signals then pass via bipolar, horizontal, and amacrine cells to RGCs, which generate action potentials. Visual information is transmitted via nerve impulses to the thalamic and then to cortical centers [23,24]. It should also be noted that ocular phylogeny is highly dependent on the physiological regulation of ion channels. Thus, photoreceptor channels control phototransduction; epithelial and endothelial channels in the cornea and ciliary body regulate transport; lens channels maintain transparency and homeostasis; disruption or dysfunction of the channels can lead to retinal dystrophy, glaucoma, cataracts, and dry eye syndrome [27]. Finally, the tear film maintains epithelial homeostasis on the ocular surface and provides a stable, transparent interface essential for tissue health and optical quality [23,24].

## 3. Cellular Therapies in Ophthalmology

In the field of ophthalmology, cell therapies represent an innovative therapeutic approach for treating a wide range of eye diseases, offering hope for the regeneration of damaged tissue and the restoration of visual function [16,28]. They are defined as therapeutic interventions involving the use of living cells to restore the function or structural integrity of the eye, which has been damaged due to pathological conditions, injuries, or age-related degenerative processes [15,29]. The significance of cell therapies lies in their ability to offer solutions to conditions for which existing conventional therapies have limited effectiveness, opening up new prospects in the field of regenerative ophthalmic medicine [28]. Among the most essential ophthalmological conditions that are the subject of research and application of cell therapies is AMD. This progressively evolving disease affects central vision. IRDs, such as retinitis pigmentosa and Stargardt disease, are characterized by genetically determined degeneration of photoreceptors and other cell types in the retina, leading to gradual loss of visual function [16,29,30]. Corneal pathologies, which may be caused by traumatic injuries, infectious complications, or chemical burns, can be treated with cell therapies aimed at regenerating the epithelial, stratum corneum, or endothelium of the cornea, as is the case in limbal stem cell deficiency (LSCD) [15,31]. Although glaucoma is a degenerative neuropathy that leads to vision loss, the research approach to cell therapies focuses mainly on protecting the ganglion cells of the retina [15,16]. In addition, DR, a common complication of diabetes mellitus that can lead to blindness, is also being studied for the development of cell therapies aimed at both vascular and neuronal protection of retinal tissue [15,16,29].

Terminology and regulatory note: In this review, cell-based therapy means any therapeutic use of living cells (e.g., differentiated corneal epithelial sheets, RPE monolayers, progenitors, or stem cells). Stem-cell therapy is a subset of cell-based therapy that uses cells with self-renewal and multilineage potential (e.g., ESCs, iPSCs, MSCs). Regulatory classification and oversight can differ between these categories (e.g., somatic cell/tissue-engineered products vs. stem-cell-based products), so we do not use ‘cell-based’ and ‘stem-cell’ interchangeably.”

### 3.1. Types and Strategies of Cell Therapies

**Figure 2:**
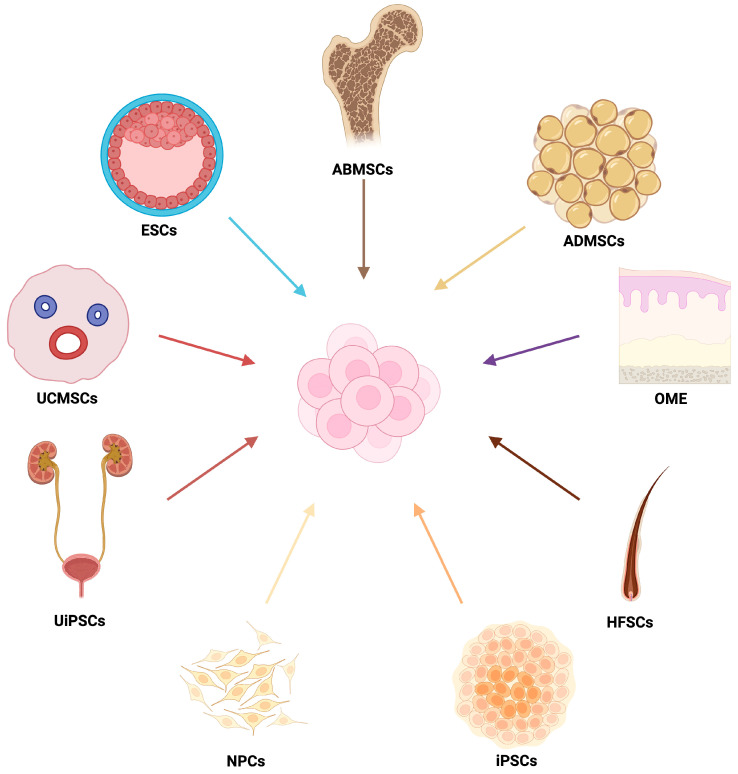
Stem-cell reservoirs leveraged for ocular therapy. Adult tissue-derived sources include autologous bone-marrow mesenchymal stem cells (ABMSCs), bone marrow-derived MSCs (BM-MSCs), adipose-derived MSCs (ADMSCs), hair-follicle stem cells (HFSCs), lens epithelial stem/progenitor cells (LECs), limbal stem cells (LSCs), neural precursor cells (NPCs), trabecular meshwork stem cells (TMSCs), oral mucosal epithelium (OME), and retinal progenitor cells (RPCs). Perinatal sources comprise umbilical cord MSCs (UCMSCs) and Wharton’s jelly-derived MSCs (WJ-MSCs). Pluripotent sources include embryonic stem cells (ESCs), induced pluripotent stem cells (iPSCs), and urine-derived iPSCs (UiPSCs) (created by https://www.biorender.com/, accessed on 5 September 2025).

Modern cell therapies can be classified into subcategories, such as stem cell therapies based on (i) autologous (the patient’s cells) and allogeneic (donor cells) [32], (ii) genetically modified cells, (iii) combine cellular encapsulation and release of extracellular vehicles (EVs) such as transcription factors, microRNA and membrane proteins [33,34,35,36,37]. The stem cells can be taken from different tissues (Figure 2). The choice between types of cells significantly affects aspects such as immunocompatibility, scalability, and logistics. There will be a discussion about this below [32,38,39].

Modern cell therapies in ophthalmology employ a range of strategies to restore vision, tailored to the cell type and disease in question. At their core, these therapies harness the regenerative capacity of stem cells or the functional activity of specialized cells to replace or support the patient’s own cells: cell replacement, paracrine and neuroprotective effects, tissue engineering and scaffolds, and immune considerations. Many approaches to cell therapy seek to physically replace cells that have died. For example, in AMD, the focus is on transplanting new RPE cells; in corneal endothelial failure, on injecting new endothelial cells. PSCs (either embryonic stem cells or iPSCs) can be directed in the lab to become the desired ocular cell type (RPE, photoreceptor, corneal cell, etc.), which is then delivered to the patient [40,41]. These transplanted cells can potentially integrate into the tissue structure—lining up as a new RPE layer under the retina or attaching to the inner cornea—and take over the job of the lost cells. In some cases, such cells are delivered on a scaffold or membrane to help them align and survive (as with RPE patches). Successful integration means the new cells perform the needed functions: RPE cells, for instance, would support and nourish photoreceptors, clear waste, and maintain the retina’s health, thereby stabilizing or improving vision [41,42]. Not all benefits of cell therapy require permanent integration. Certain treatments use stem cells (like MSCs) for their ability to secrete neurotrophic and anti-inflammatory factors [43,44,45]. When injected into the eye, these cells can act as “mini-drug factories”, releasing growth factors, cytokines, and vesicles that protect retinal neurons from ongoing degeneration and encourage tissue repair. This strategy is being explored for optic nerve injuries and early-stage retinal diseases—essentially as a biologic therapy to create a more regenerative environment inside the eye. Even if the transplanted cells do not survive long-term, the beneficial molecules they secrete may catalyze healing or prolong the life of the patient’s remaining cells [43,45]. Modern cell therapy often intersects with bioengineering. Rather than injecting a suspension of cells, scientists sometimes grow cells on biocompatible scaffolds [46] or membranes to create a small tissue that can be implanted [47,48]. The RPE patch for AMD is one example: a monolayer of RPE cells grown on a thin support that surgeons place under the patient’s retina. Similarly, sheets of cultivated corneal epithelium can be transplanted onto scarred corneas (an approach used for ocular surface burns). These structures help cells maintain the proper orientation and density, increasing the chances that they will function correctly after transplantation. They effectively act as a replacement part for the eye, engineered in the lab from the patient’s or a donor’s cells [47,48]. The immune-privileged status of the eye means that in many cases, allogeneic cells (from donors) can survive without instant rejection, especially if placed in the subretinal space or anterior chamber, which lack a direct blood supply [49,50]. Nonetheless, immune compatibility is still considered—for example, iPSCs allow autologous transplants (from the patient’s own cells), which virtually eliminate rejection risk. Researchers are also exploring gene-editing to “cloak” cells from the immune system or using transient immunosuppression around the time of cell delivery. The goal is to achieve durable engraftment of new cells with minimal side effects [49,50].

### 3.2. Parthenogenetic Stem Cells

Parthenogenetic embryonic stem cells (hPG ESCs or pESCs) are derived from the development of blastocysts in vitro following parthenogenetic activation of human oocytes, a process that is one of the main and most useful methods of producing embryonic stem cells (ESCs) [51]. These cells exhibit typical ESC morphology and express the expected markers, such as SSEA-4, TRA-1-60, and Oct-4, while their parthenogenetic origin is confirmed by gene imprinting data (gene imprinting). hPG ESC cell lines are an attractive alternative and important source of histocompatibility cells and tissues for future cell therapies, as they are considered mainly homozygous for major histocompatibility complex (MHC/HLA) alleles (MHC/HLA), thus offering the possibility of immune matching in a significantly larger number of patients compared with conventional ESCs [52]. These cells exhibit typical ESC morphology and express the expected markers, such as SSEA-4, TRA-1-60, and Oct-4, while their parthenogenetic origin is confirmed by gene imprinting data (gene imprinting). hPG ESC cell lines are an attractive alternative and important source of histocompatible cells and tissues for future cell therapies, as they are considered mainly homozygous for major histocompatibility complex (MHC/HLA) alleles (MHC/HLA), thus offering the possibility of immune matching in a significantly larger number of patients compared with conventional ESCs [53]. Challenges include managing immune compatibility, as reductive recombination during parthenogenetic activation can restore partial heterozygosity at loci such as MHC, which should not be overlooked. Other biological challenges relate to potential karyotype instability, such as the occurrence of abnormalities (e.g., chromosomal translocations) observed in the hPES-2 line after more than 100 passages, as well as with the unbalanced expression or loss of imprinting genes due to monogenic origin, which is potentially associated with an increased risk of tumors. Finally, ethical issues related to research on human parthenogenetic stem cells are part of the general debate on egg donation and embryonic stem cell research [54,55].

### 3.3. Mechanisms of Stem Cell Integration in the Eye

Stem-cell integration in the eye is governed by a coordinated sequence of homing, anchorage to specialized extracellular matrices, survival within immune-modulated niches, and finally, synaptic or epithelial coupling that restores tissue function. On the ocular surface, limbal epithelial stem cells (LESCs) respond to chemokine gradients—most notably SDF-1/CXCL12 engaging CXCR4—to traffic toward the limbal niche and resist premature differentiation; successful grafts recapitulate this axis to promote centripetal resurfacing of the cornea [56]. Within this niche, Wnt–PAX6 programs (including WNT7A and WNT6) maintain stemness and corneal identity, while the tissue’s biomechanics tune Hippo–YAP activity: softer, physiologic limbal stiffness drives nuclear YAP and preserves a regenerative phenotype, a mechanism increasingly leveraged in engineered grafts and biomaterials [57]. Stable epithelialization then depends on integrin-mediated adhesion to basement-membrane laminins (e.g., laminin-511/521) and collagen IV; these cues steer proliferation and sheet migration after transplantation [58]. In this surface context, mesenchymal stromal cells (MSCs) chiefly contribute via paracrine immunomodulation and trophic support (e.g., anti-inflammatory cytokines, extracellular vesicles) that enhance host-cell survival and matrix remodeling, whereas epithelial stem-cell-based constructs provide a direct structural replacement of the corneal surface. In the outer retina, integration hinges on re-establishing the photoreceptor–RPE unit and rebuilding first-order synapses. Human ESC/iPSC-derived RPE grafts integrate as a polarized monolayer that supports photoreceptors by reinstating the αvβ5-integrin→MFG-E8→MerTK pathway for circadian outer-segment recognition, cup formation, and actin–PI3K–FAK-driven phagocytosis—molecular steps tightly linked to photoreceptor survival after surgery [59]. Adjunctive ROCK inhibition (e.g., Y-27632) improves graft viability and adhesion in preclinical primate and rodent studies and is now commonly considered in RPE sheet or strip formulations [60]. Because the subretinal space is immune-reactive rather than immune-privileged in disease, long-term survival additionally depends on modulating HLA expression: MHC-II (CIITA) knockout iPSC-RPE suppresses leukocyte infiltration and rejection in non-human primates, and contemporary strategies layer non-classical HLA (E/G) or “hypoimmunogenic” editing to blunt T/NK-cell recognition [61]. By contrast, MSCs delivered intravitreally or subretinally are not expected to form a pigmented epithelium or execute outer-segment phagocytosis; instead, their benefits are indirect, arising from neurotrophic, anti-apoptotic, and anti-inflammatory signaling that stabilizes the host photoreceptor–RPE complex and limits secondary degeneration. For photoreceptor precursors, structural incorporation into the outer nuclear layer must be matched by molecular assembly of the ribbon synapse. Recent work shows that trans-synaptic organizers such as ELFN1 (presynaptic in rods) and LRIT3 (cone-biased) recruit and stabilize the postsynaptic mGluR6/TRPM1/nyctalopin complex on ON-bipolar cells; disrupting either partner degrades synaptic enrichment, whereas restoring LRIT3 or proper mGluR6 glycosylation rescues function—mechanisms directly relevant to donor–host synaptogenesis after transplantation [62]. Beyond laminar placement and ribbon-synapse assembly, grafted RPE must also re-establish metabolic and structural support to stabilize photoreceptors. A correctly polarized monolayer restores daily outer-segment clearance via the αvβ5–MFG-E8–MerTK axis, couples phagocytic flux to actin–PI3K–FAK signaling, and sustains the visual cycle through retinoid recycling, β-oxidation of phagocytosed discs, and lactate shuttling; apical microvilli re-form intimate contact with outer segments while tight junctions (e.g., ZO-1/CRB complex) re-establish barrier function and vectorial transport [59]. Together with ELFN1/LRIT3→mGluR6/TRPM1-dependent synaptogenesis in ON-bipolar circuits, these processes couple cellular integration to circuit function and long-term photoreceptor survival [59,62].

By contrast, inner-retinal and optic-nerve repair add formidable physical and intrinsic barriers. The internal limiting membrane (ILM)—a laminin/collagen-rich basement membrane over Müller endfeet—prevents transplanted retinal ganglion cells (RGCs) from entering the parenchyma; enzymatic disruption or surgical/laser “photodisruption” of the ILM disperses grafts, enables neurite ingrowth into the inner plexiform layer, and is emerging as a prerequisite step for RGC integration in ex vivo and in vivo models [63]. Even after somatic integration, axon extension through the optic nerve requires reactivating growth programs normally silenced after development: combinatorial manipulation of cell-intrinsic pathways (PTEN/mTOR, SOCS3–JAK-STAT with CNTF) and inflammation-derived cues such as oncomodulin—now linked to its receptor ArmC10—enhances RGC survival and long-distance regeneration, pointing to adjuvants that could be paired with stem-cell replacement to reconnect central targets [64]. In these inner-retinal settings, MSCs again act predominantly as microenvironmental modulators (neurotrophic support, extracellular matrix remodeling, and immune regulation), whereas cell-replacement strategies (e.g., RPE or neuronal precursors) must physically integrate—overcoming matrix barriers like the ILM and reinstating polarity, synapses, or axon growth—to restore circuit function [56].

### 3.4. Emerging Immunomodulatory Strategies in Ocular Cell Therapy

Ocular cell transplantation, while benefiting from the eye’s relative immune privilege, still faces immune-mediated graft loss. Recent clinical and preclinical studies, therefore, employ short-term immunosuppression around the time of transplant to promote engraftment. For example, Gowrishankar et al. found that in neural and retinal stem cell trials, multi-agent regimens (tacrolimus, mycophenolate mofetil, and steroid taper) were commonly used for only a few months, and grafts remained viable for months to years after stopping therapy [65]. Likewise, in retinal pigment epithelium (RPE) transplant trials, immunosuppression is typically started about one week pre-operatively and continued for several months post-operatively [47]. Local delivery of steroids (e.g., intravitreal triamcinolone) has also been used in some retinal studies to provide targeted immunosuppression [65]. These protocols (sometimes referred to as “transient” or perioperative immunosuppression) have generally enabled long-term graft survival. For instance, reported RPE implants and limbal allografts often required only perioperative therapy, yet remained intact without signs of acute rejection [65]. Overall, a consensus is emerging that multi-drug, short-duration immunosuppressive courses can mitigate host rejection and allow durable ocular graft survival while minimizing systemic exposure [65].

#### 3.4.1. Gene-Engineering of Immune-Evasive Grafts

A complementary strategy is to “cloak” graft cells via genetic engineering. It has been demonstrated that knocking down or modulating HLA genes in donor cells can significantly reduce host immune activation [66]. For example, CRISPR deletion of the class II transactivator (CIITA) in iPSC-derived RPE abolished MHC-II expression and profoundly suppressed leukocyte infiltration and rejection in non-human primate models. Similarly, Kim et al. engineered human iPSCs with targeted knockouts of HLA-A, HLA-B, and HLA-DRα, yielding “universal” hypoimmunogenic cells. The resulting triple-KO iPS cell clone lacked detectable HLA expression even after interferon-γ stimulation and failed to elicit proliferation of host memory T cells in vitro [66]. These findings indicate that extensive removal of classical HLA loci can create donor cells that largely evade T cell recognition. Other approaches layer in non-classical HLA or immune checkpoint molecules: for example, enforced expression of HLA-E or HLA-G has been proposed to inhibit NK-cell killing of HLA-null cells. In short, the emerging field of immune gene-editing is actively developing multi-gene “hypoimmunogenic” cell lines for allogeneic ocular grafts. These engineered cells are designed to be “invisible” to host adaptive immunity, potentially obviating the need for prolonged immunosuppression [66].

#### 3.4.2. Biomaterial Scaffolds and Engineered Niches for Local Immune Modulation

Biomaterial scaffolds and delivery platforms can also be exploited to modulate local immunity in the eye. On the ocular surface, for instance, amniotic membrane (AM) transplants are known to exert anti-inflammatory effects: AM used as a scaffold for limbal stem cell grafts “exhibit remarkable anti-inflammatory and immunomodulatory effects” that help suppress rejection [67]. Likewise, synthetic hydrogels or nanofiber scaffolds loaded with mesenchymal stem cells (MSCs) or immunomodulatory drugs have been shown to dampen corneal inflammation (e.g., by reducing MMP-9, iNOS, and VEGF expression in alkali burn models) [67]. In the subretinal space, RPE carriers are being designed with immunomodulation in mind. Current RPE scaffolds (e.g., collagen or peptide hydrogels, polymer membranes) not only support cell delivery but can be engineered to control immune responses. For example, microporous peptide-crosslinked hydrogels and 3D-printed Bruch’s membrane analogs have been shown to influence macrophage polarization in vitro [47]. Future designs aim to incorporate anti-inflammatory agents or to tune degradation profiles to actively reduce microglial activation and lymphocyte recruitment in vivo [47]. In effect, these biomaterial niches function as localized immunoregulatory microenvironments [68]: By providing sustained release of drugs or instructive topography and chemistry, they can bias host innate immunity toward tolerance. In combination, these engineered scaffolds and encapsulation devices (for example, the CNTF-releasing NT-501 implant) demonstrate that biomaterials can serve as both physical support and localized immune modulators in ocular cell therapy [67].

## 4. Cell Therapy

### 4.1. Autologous Stem Cell Therapy

**Figure 3:**
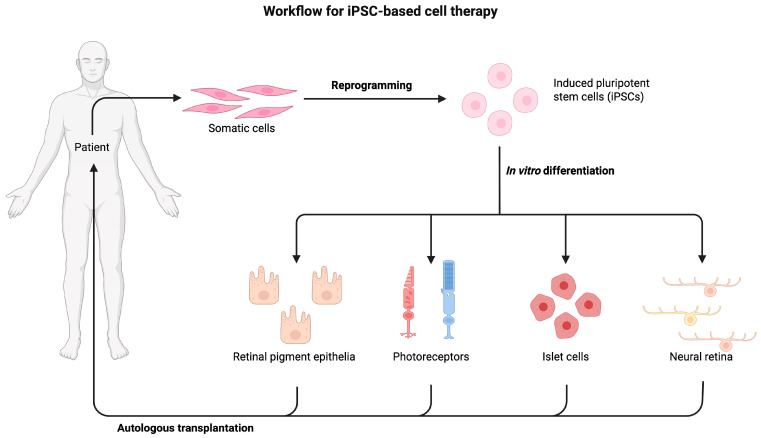
Autologous iPSC-derived retinal cell therapy workflow for ocular diseases (created by https://www.biorender.com/, accessed on 5 September 2025).

The primary difference between autologous and allogeneic stem cell therapies stems from the origin of the cells, which, in turn, determines their immunological compatibility and potential for immune response. Autologous stem cell therapy involves the transplantation of a patient’s cells, which are harvested, expanded, and then reintroduced into the same individual [32,69]. The principal advantage of this approach is the near elimination of concerns regarding immune rejection, as the transplanted cells are genetically identical to the recipient, thus obviating the need for immunosuppressive drugs [32]. This immune compatibility is highly desirable for long-term graft survival. However, autologous therapies typically entail a more time-consuming and costly process due to the need for individual cell harvesting, reprogramming in the case of iPSCs (Figure 3), and expansion, which can delay treatment [32]. Furthermore, there is a risk of tumorigenicity if the cell products contain residual undifferentiated PSCs. Additionally, for adult stem cell sources like MSCs, the limited number of cells obtainable from elderly or severely ill patients can pose a challenge for autologous transplantation [69,70]. Cases of severe vision impairment have also been reported following intravitreal injection of autologous adipose-derived MSCs for AMD, underscoring the critical need for comprehensive regulatory oversight and patient safety [71,72].

### 4.2. Allogeneic Stem Cell Therapy

Allogeneic stem cell therapy uses cells from a donor instead of the patient, which is different from autologous approaches [32]. This strategy has a lot of benefits, especially when it comes to scalability and being able to use it right away. In this case, cells can be made in large quantities, frozen, and stored, which makes it possible to use them in clinical settings right away [72,73]. So, allogeneic therapies are easier to obtain and do not require as much planning as patient-specific autologous treatments. But the biggest problem with allogeneic transplantation is that the recipient’s immune system might reject the graft, which usually means that the recipient needs to be given systemic immunosuppression to keep the graft alive [32,74]. The eye’s natural immune-privileged environment, which is mostly due to the blood–ocular barrier, can greatly lower the risk of rejection for treatments given directly to the eye. Additionally, some allogeneic cell types, especially MSCs, have immune-modulating properties and are less likely to cause an immune response. This may lower the risk of immune rejection [72]. Ethical issues are also important, especially when it comes to allogeneic therapies that come from hESCs because they come from embryos. When using autologous iPSCs or MSCs, these kinds of ethical problems do not usually come up [74]. Also, therapies that come from pluripotent sources like hESCs have a high risk of causing tumors, so they need to be carefully evaluated and monitored.

Despite ocular immune privilege, immune-mediated graft compromise has been documented in recent eye cell-therapy trials, including RPE implants (clinical rejection and lab immune activation) [75,76] and allogeneic limbal grafts [77,78]. These data justify peri-operative immunosuppression protocols, HLA matching/engineering, and monitoring for subclinical rejection in future studies.

A comparison of stem cell types and their applications in ophthalmology is presented in Table 1 and Table 2, which compare recent interventional clinical trials in 2023–2025.

### 4.3. Combine Cellular Encapsulation and Release EVs

Advanced strategies in ophthalmology—notably encapsulated cell technology (ECT) and EVs—aim to overcome the limits of conventional care by providing targeted, long-lasting therapy for diseases of the retina and anterior segment. ECT. ECT is designed to release therapeutic proteins continuously inside the eye, right where they are needed. Devices such as the NT-501 implant house genetically modified cells—often RPE cells—within a semipermeable polymer capsule that lets proteins diffuse out while protecting the cells from the host immune system [87,95]. In practice, this means sustained delivery of agents like CNTF without repeated injections, which can improve comfort and adherence. Because the implant is retrievable, clinicians can remove it if needed, a safety feature not available with permanent approaches such as some gene therapies [96]. Clinically, ECT has been studied in chronic retinal disorders, including GA in AMD, where it has shown dose-dependent increases in retinal thickness and stabilization of visual acuity. It is approved for idiopathic macular telangiectasia type 2 (MacTel type 2) and continues to be evaluated in RP for its potential to slow photoreceptor loss by sustaining neurotrophic support [97,98]. EVs—exosomes and microvesicles—are nanoscale packets released by many cell types, including MSCs and human amniotic epithelial cells (hAECs) [95,96]. They act as natural couriers, carrying proteins, lipids, mRNAs, and microRNAs to recipient cells via endocytosis, membrane fusion, or receptor-ligand interactions [99]. EVs are attractive therapeutics because they show low immunogenicity, minimal toxicity, and can cross barriers such as the blood-retina and blood-brain barriers—useful when cell therapies raise concerns about rejection or tumorigenesis. In diabetic retinopathy (DR), MSC-derived EVs reduce apoptosis, inflammation, and pathological angiogenesis; notably, hUCMSC-EVs deliver miR-30c-5p to dampen NF-κB signaling [100,101,102]. In DED, hAEC-EVs promote corneal epithelial proliferation and migration and lower pro-inflammatory cytokines [34,98]. For corneal injury and fibrosis, MSC-derived exosomes aid wound healing and inflammation control, largely through microRNA cargo [98]. EVs are also being explored for neuroprotection in glaucoma and optic-nerve injury, with encouraging evidence for retinal ganglion cell survival and axonal regeneration [38,103]. Their ability to deliver therapy locally and safely positions them as powerful complements to existing options—and promising foundations for the next generation of ophthalmic care. So, both ECT and EV-based therapies bring the field closer to precise, sustained, and biocompatible treatment.

### 4.4. D Bioprinting for Ocular Therapies

Three-dimensional (3D) printing and bioprinting represent transformative technologies that are increasingly reshaping ophthalmology and eye care, moving towards the development of personalized therapeutic strategies [104,105]. These additive manufacturing processes enable the precise, layer-by-layer fabrication of complex structures by depositing various biomaterials, functional elements, and living cells into predefined 3D geometries, often based on digital models derived from clinical images such as CT or MRI scans [104,105,106].

In corneal tissue engineering, 3D bioprinting is actively explored to create artificial corneal structures that effectively mimic the native human cornea, particularly the stroma, which accounts for 80% of corneal thickness [104,105,106]. Researchers have successfully bioprinted artificial corneal stromal equivalents using bio-inks containing encapsulated human corneal keratocytes within collagen-based or alginate/collagen composite bio-inks [104,106,107]. These constructs have demonstrated high cell viability, exceeded 90% immediately post-printing, and maintained 83% viability after seven days [107]. Efforts are also underway to bioprint other corneal layers, including the epithelium, using human corneal epithelial cells in alginate/gelatin inks, with some success in achieving high transparency and cell viability. Similarly, genetically modified human corneal endothelial cells have been bioprinted onto supportive membranes, showing promising transparency and cell activity in both in vitro and in vivo rabbit models, suggesting a potential alternative to conventional corneal transplantation for endothelial diseases [105]. Key challenges in corneal bioprinting include achieving optimal transparency and mechanical properties akin to the native cornea, as well as reproducing its complex multi-layered structure [104,107]. Adjustments in printing parameters, such as nozzle diameter and bio-ink viscosity, are crucial to balance print accuracy with mechanical stability and cellular behavior [107].

Retinal tissue engineering presents a more formidable challenge due to the retina’s intricate neural complexity and layered organization. Despite this, researchers are developing multilayered retinal models that aim to replicate the RPE and photoreceptor layers [105,108]. Techniques such as microvalve-based bioprinting and laser-assisted 3D printing have enabled the fabrication of structures with RPE and photoreceptor cells, demonstrating good cell viability and cytocompatibility, with the long-term goal of creating autologous retinal cell grafts for degenerative diseases like retinitis pigmentosa and AMD [105,108]. Studies have shown successful printing of retinal ganglion cell neurons and glial cells with adequate viability, with printed glial cells retaining their growth-promoting capabilities [109]. The stiffness of bioprinted scaffolds has been demonstrated to be similar to that of the native retina, indicating suitable mechanical environments for cellular function and differentiation [105,108]. Beyond tissue regeneration, 3D printing finds diverse applications in ophthalmology. It facilitates the creation of personalized implants and prostheses, such as customized orbital implants for facial trauma and ocular prostheses, which are patient-specific, enhancing fit, comfort, and surgical outcomes [104,107]. Drug delivery systems are also being revolutionized, allowing for the production of customized oral dosage forms, conjunctival drug-releasing patches, and contact lenses for sustained and efficient drug release (e.g., nanoparticle-modified lenses for glaucoma), overcoming limitations of traditional eye drops like non-adherence and systemic side effects [104,105,107,110]. Furthermore, 3D printed anatomical models and surgical guides are invaluable tools for complex ophthalmic procedures, aiding in preoperative planning, patient education, and surgical training by providing a detailed 3D view of anatomical relationships [107,111]. The selection of appropriate bio-inks and printing techniques is paramount for successful outcomes [105]. Commonly used bio-inks include naturally derived polymers like collagen, alginate, gelatin, and hyaluronic acid, chosen for their similarity to the human extracellular matrix and inherent bioactivity [107,112]. Composite hydrogels, combining natural and synthetic materials, are often employed to balance mechanical strength with a cell-friendly environment [107,112]. Various 3D printing techniques are utilized, including extrusion-based bioprinting for continuous filament deposition, compatible with a wide range of bio-ink viscosities [106,107,113], inkjet-based bioprinting for precise picoliter droplet dispensing, suitable for low-viscosity materials and high resolution [112,114] and light-assisted bioprinting, such as Digital Light Processing or Two-Photon Polymerization, offering rapid printing speeds and high resolution, though material selection is limited to photopolymerizable substances [112,114]. Despite significant advancements, several challenges continue to impede the widespread clinical application of 3D bioprinting in ophthalmology. These include regulatory complexities, the need for more comprehensive long-term studies, and difficulties in sourcing sufficient and diverse regeneration-competent cells [105,111]. Achieving the structural and functional intricacy of native ocular tissues remains technically demanding, and ensuring long-term cell viability and integration poses ongoing concerns. Additionally, the high costs and interdisciplinary nature of the field may limit scalability. Nevertheless, 3D bioprinting holds considerable promise for advancing personalized approaches to eye tissue regeneration, drug delivery, and surgical intervention [105,108].

## 5. Imaging Tools for Monitoring Graft Survival and Integration

Newer imaging modalities are crucial for achieving non-invasive, high-resolution in vivo monitoring of cellular graft survival and integration in ophthalmology, often surpassing the limitations of histology [115,116]. Optical Coherence Tomography (OCT) serves as a foundational tool, providing high-resolution, cross-sectional, and volumetric (3D) structural images of the retina and choroid, enabling crucial, detailed visualization of morphological changes and high-precision retinal thickness measurements over longitudinal study periods [117]. Moreover, OCT allows for the direct visualization of transplanted stem cells as hyperreflective signals and is frequently integrated into surgical procedures to provide real-time guidance during subretinal cell delivery [115,116]. To elevate resolution to the cellular level, Adaptive Optics (AO) systems are incorporated into OCT instruments (AO-OCT) to correct the refractive aberrations inherent to the eye [115,116]. AO enables diffraction-limited lateral resolution, a necessity for visualizing fine structures like the photoreceptor mosaic and for analyzing detailed cellular structures following the transplantation of induced pluripotent stem cell (iPSC)-derived retinal organoids. However, since conventional OCT cannot definitively distinguish transplanted donor cells from native host tissue, Multimodal Imaging (MMI) platforms are essential for a comprehensive evaluation of graft destiny and function [115]. For instance, OCT Angiography (OCTA), a high-resolution, non-invasive functional extension of OCT, allows for the selective visualization of blood flow in the choriocapillaris and has been successfully utilized to document the reperfusion and vascular re-anatomization necessary for the survival of translocated autologous RPE-choroid grafts [118]. Furthermore, advanced preclinical MMI systems, such as the combined Photoacoustic Microscopy (PAM), OCT, and Fluorescence Microscopy (FM) (PAOFM), leverage biocompatible nanosensors—like ultraminiature gold nanoparticle clusters (GNCs) and semiconductor quantum dots—to non-invasively track the 3D distribution, migration, and viability of transplanted hiPSC-RPE cells over periods extending up to six months with exceptional spatial resolution and sensitivity [119,120,121]. This integration of structural, functional, and molecular tracking capabilities confirms that multimodal approaches, particularly those incorporating high-resolution methods like AO-OCT and contrast-enhanced techniques, are vital for assessing the complex processes of graft survival and integration required for modern cellular therapies [116,117].

## 6. Biomarkers and Functional Endpoints in Retinal Cell Transplantation

### 6.1. Biomarkers of Graft Integration and Rejection

Recent retinal transplant studies emphasize the need for objective biomarkers to track graft survival, synaptic integration, and immune status. In vivo imaging modalities (e.g., spectral-domain OCT, fundus autofluorescence, scanning-laser ophthalmoscopy) can quantify transplant parameters such as graft size, laminar organization, and localization. For example, multimodal imaging in rodent models has been used to score graft size, on-target placement, lamination, hemorrhage, and atrophy, with image grades correlating tightly with histology [122]. Similarly, electrophysiological mapping (e.g., multifocal ERG or superior colliculus recordings) provides functional evidence of donor photoreceptor activation and host connectivity. Lin et al. demonstrated that transplanted human retinal organoids elicited point-to-point visual responses in the superior colliculus, congruent with graft location [123], confirming synaptic integration. In short, imaging and physiological measures are emerging as quantifiable integration biomarkers [122].

In contrast, biomarkers of immune rejection remain underdeveloped. The eye’s partial immune privilege slows rejection, but transplanted iPSC-derived RPE still provokes immune responses days to months post-operation [124,125,126]. Candidate rejection markers include complement fragments (e.g., C3a, C5b-9), pro-inflammatory cytokines (IL-1β, IL-6, TNF-α, IFN-γ), and donor-specific antibodies, analogous to other grafts. For instance, complement activation is a key feature in xenograft rejection, yet complement components are not routinely assayed in ocular transplant patients [127]. Similarly, tear or aqueous biomarkers (e.g., elevated IL-6 or IL-17 in corneal graft rejection) have been proposed but not validated for retinal grafts. In practice, most studies rely on indirect surrogates (imaging or visual function) and intensive immunosuppression rather than definitive immune assays. As such, identifying specific molecular or cellular rejection biomarkers is an active research goal, and future protocols may incorporate serial immune monitoring alongside structural measures.

### 6.2. Limitations of Best-Corrected Visual Acuity (BCVA)

BCVA has long been the standard clinical endpoint in retinal trials, but it has important shortcomings. First, BCVA measures only high-contrast central acuity and often remains preserved until late stages of disease. In many degenerations (e.g., early AMD or mid-stage inherited dystrophies), patients lose extensive peripheral or low-contrast vision long before BCVA changes. Expert consensus notes that VA exhibits high test–retest variability and poor sensitivity to early disease [128]. For example, Schaal et al. observed that it is “unrealistic to use visual acuity as a clinical trial endpoint in non-exudative AMD because vision loss takes many years to develop” [128]. Similarly, a choroideremia trial analysis ranked standard BCVA lowest among functional measures in terms of sensitivity; microperimetry and contrast tests were far more responsive to progression [129]. In practice, subjects may have stable or only minimally changed BCVA even as retinal sensitivity or visual field worsens.

Moreover, BCVA does not capture real-world visual quality. In recovered patients (e.g., after uveitis or retinal detachment), BCVA may return to normal levels while patients still report significant visual complaints. Studies of Vogt–Koyanagi–Harada (VKH) disease found that visual acuity “poorly reflects accurate visual functions in real life” [130]: even after VA recovery, patients often have persisting deficits in color vision, contrast tasks, or electrophysiological responses [130]. In summary, BCVA is a coarse, late-stage indicator that misses early and subtle changes. Its ceiling effect (can not improve beyond 20/20) and floor effect (poor resolution at very low vision) further limit its usefulness as a primary trial endpoint.

### 6.3. Alternative Functional Vision Metrics

To address BCVA’s limitations, additional metrics are increasingly used as trial outcomes (Table 3). These functional vision tests capture aspects of vision more relevant to daily life and earlier disease changes. Examples include the following: (i) Contrast Sensitivity (CSF): Tests the ability to discern low-contrast patterns. Contrast sensitivity often declines well before BCVA drops. CSF correlates better with patient-reported visual difficulties and quality of life. In practice, contrast sensitivity testing has revealed deficits in “recovered” patients with normal VA and is considered more sensitive to subtle visual dysfunction [128]. (ii) Microperimetry: Measures localized retinal sensitivity across the macula under fixation control. Unlike VA, microperimetry can map functional scotomas and quantify parafoveal cone function. It has proven highly effective in retinal disease trials (AMD, inherited dystrophies, etc.) [128]. For example, experts found microperimetry to be the single most important functional measure predicting disease progression in choroideremia, outperforming BCVA [128]. Microperimetry is now commonly used as an endpoint in modern retinal trials (e.g., gene therapies for IRDs) precisely because it sensitively tracks localized loss that BCVA misses. (iii) Electrophysiology (ERG/VEP)*:* Objective recordings of retinal or cortical responses can reveal global function that behavior-based tests cannot. Full-field ERG assesses overall photoreceptor activity, while multifocal ERG maps regional function. Visually evoked potentials (VEPs) or superior colliculus recordings (in animals) measure downstream signal transmission. These tools have shown value in graft studies: for instance, after photoreceptor transplant in rats, point-to-point retinal projections could be detected on superior colliculus electrophysiological maps [123]. In clinical terms, ERG can document restoration of outer retinal currents even when VA is unchanged. Importantly, electrophysiology is not influenced by patient effort or chart familiarity, providing a reproducible complement to subjective tests.

Collectively, these functional metrics provide a more nuanced view of vision than BCVA alone. Contrast sensitivity and microperimetry detect early or low-contrast losses [128], while electrophysiological tests objectively quantify retinal and neural function. By combining high-contrast VA with these additional measures, trials can better characterize visual gains or losses. In summary, the field is moving beyond BCVA as the sole endpoint: modern studies now routinely incorporate contrast sensitivity curves, microperimetric sensitivity maps, and ERG-based assays to capture the multifaceted nature of visual function.

## 7. Applications in Ophthalmologic Diseases

### 7.1. Corneal Diseases

There are currently several region-specific approved cell therapy products for corneal diseases—Holoclar^®^ (EU), Nepic^®^ (Japan), and Ocural^®^ (Japan)—but these approvals do not imply global standard-of-care status. Additionally, there are ongoing clinical trials that highlight the importance of cell-based therapy.

Holoclar^®^ is a cell-based tissue-engineered therapy that replaces epithelial cells in a damaged cornea and was the first stem cell product to receive marketing authorization in the EU [88]. It is designed to treat LSCD, a condition resulting from severe eye burns (chemical or physical) that destroy LSCs, leading to vision loss due to conjunctival cell invasion, neovascularization, inflammation, stromal scarring, and corneal opacity [88]. The treatment involves taking a small biopsy of the patient’s own LSCs from their unaffected eye, followed by ex vivo expansion. The manufacturing process, which includes initial culture with irradiated mouse feeder cells (3T3-J2) and cryopreservation as an intermediate cell bank, ensures the maintenance of holoclone content in the final product [136,137]. The final product is administered by grafting the cells, which are cultured on a fibrin matrix, into the injured eye to restore the LSC population and regenerate a normal, transparent corneal surface. A phase IV clinical trial (NCT02577861) of Holoclar^®^ assessing the efficacy and safety of this therapy one year after the first treatment was completed (no results published yet), but clinical experience has shown a positive correlation between the percentage of holoclones in the culture and clinical success, and long-term follow-up has confirmed the absence of tumorigenicity in vivo [138].

Similarly, Nepic^®^ is an autologous cultured corneal epithelium and was approved in 2020 as the first regenerative medicine product in the ophthalmology field in Japan. It is also developed for the treatment of LSCD. The manufacturing process of Nepic^®^ involves separating corneal epithelial cells from a small piece of the patient’s own limbal tissue, culturing them with feeder cells (3T3-J2 cells from a banked source) to promote proliferation, and then forming a sheet on a temperature-responsive culture dish. This cell sheet is then packaged in a preservation solution for the final product. Clinical trials in Japan achieved a corneal epithelial reconstruction success rate of 60% at 52 weeks post-transplantation, increasing to 70% at 104 weeks, a result considered comparable to other established transplantation methods [139].

While both Holoclar^®^ and Nepic^®^ are autologous, tissue-engineered therapies for LSCD, employing 3T3-J2 feeder cells, there are some key differences in their manufacturing and handling. A notable distinction is the final product form and substrate: Holoclar^®^ is administered on a fibrin matrix, whereas Nepic^®^ is manufactured as a sheet on a temperature-responsive culture dish [88,139]. Another significant difference lies in their storage and shelf-life flexibility: Holoclar^®^ incorporates a cryopreserved intermediate cell bank, which allows for a shelf-life of up to 366 days, providing options for manufacturing alignment with patient needs and even the potential for a second graft without requiring a new biopsy [88]. In contrast, Nepic^®^ cannot be cryopreserved and has a significantly shorter effective period of 60 h from the completion of sealing in its primary container, necessitating a highly coordinated, order-based production for each patient [139]. Furthermore, while Holoclar^®^ emphasizes rigorous process validation to ensure consistency across batches despite donor variability, Nepic^®^ manages patient-specific cell quality by adjusting culture conditions, such as culture duration, to suit the proliferation status of each patient’s cells, as a standardized manufacturing method would not be effective for such diverse living cell products [88,139].

In addition, a clinical trial (NCT02592330) is testing the use of autologous limbal epithelial stem cells called CALEC to combat LSCD. The cells are also cultured ex vivo from the patient’s healthy eye and then transplanted into the affected eye. Early studies have shown that CALEC has a success rate of over 90% in terms of complete or partial healing and is considered safe, with only one case of infection reported [140]. Its effectiveness in restoring corneal structure using the patient’s own cells makes it ideal for cases of unilateral damage, consistently yielding positive results for up to 18 months. Despite the clinical success of the product, challenges such as storage and preservation constraints have hindered its transition into a formally approved therapeutic agent compliant with FDA regulatory standards [140].

Another clinical trial (NCT01562002) assessed the transplantation of allogeneic bone marrow-derived MSCT compared with cultivated limbal epithelial transplantation (CLET) in patients with bilateral LSCD. The study employed a randomized, double-masked design, where CLET grafts were cultured on amniotic membrane and administered under systemic immunosuppression. At the same time, MSCT involved the infusion of donor-derived MSCs. At 6–12 months follow-up, both approaches demonstrated comparable safety and efficacy. Overall success rates ranged between approximately 76% and 86% for MSCT and 73% to 78% for CLET, with no significant differences. In vivo confocal microscopy revealed considerable improvement in central corneal epithelial phenotype in most treated eyes, and no cell-related adverse events were reported [89].

Despite the effectiveness of previous cell transplantation results, there are limitations in the availability of donor tissue for ocular surface reconstruction. To overcome this problem, in vitro expanded cultured oral mucosal epithelial cells (COMET) can be used as an alternative source of limbal stem cells [141]. Clinical studies reported success rates up to 80%, with transplanted cells expressing proliferation and corneal differentiation markers (Ki67, p63, CK3, CK12) and functioning without the need for long-term systemic immunosuppression [142]. Although COMET presents a higher angiogenic potential compared with limbal epithelial cells, no tumorigenic effects have been observed [143]. The first approved COMET-based product—Ocural^®^, was introduced in Japan in 2021, but it also has serious side effects, such as persistent epithelial defects, neovascularization, and fibrosis, while the cellular mechanisms guiding corneal-like differentiation remain poorly defined [90,144].

Building on the growing interest in stem cell-based therapies for corneal disorders such as LSCD, attention has also turned toward their potential application in other degenerative corneal conditions such as keratoconus, corneal burns, ulcers, and scars (in NCT05279157 used MSCs from adipose tissue [145,146], NCT04932629 investigated the transplantation of ex vivo cultivated allogeneic LSCs).

Beyond structural corneal disorders such as keratoconus, regenerative therapies have also gained attention in the management of ocular surface conditions like DED, where impaired epithelial healing and tear deficiency play a central role in disease progression and patient discomfort. The randomized, double-blinded Phase 2 study NCT04615455 (“AMASS”) evaluates the safety and efficacy of a single transconjunctival injection of allogeneic ASCs into the lacrimal gland of one eye in patients with aqueous-deficient DED (ADDE) associated with Sjögren’s syndrome. With 40 participants randomized 1:1 to either ASCs or placebo (CryoStor CS10 vehicle), the trial aims to determine whether lacrimal gland delivery of ASCs increases tear production and ocular comfort compared with the control. Although specific outcome data are not posted, the study has been completed.

In parallel, NCT03878628 investigates allogeneic adipose-derived MSCs administered via lacrimal gland injection for ADDE, similarly targeting tear secretion improvement and inflammation reduction. And NCT03302273 is a single-arm pilot study of topically administered corneal epithelial stem cell-derived supernatant eye drops in patients with severe DED refractory to standard treatments, delivered four times daily for 12 weeks. While neither trial has posted formal results yet, the distinction lies in their cell-derived therapeutic modality: NCT04615455 and NCT03878628 utilize cell-based lacrimal gland injections of ASCs, whereas NCT03302273 employs a cell-free topical approach using epithelial stem cell secretions for ocular surface regeneration. As part of ongoing efforts to restore corneal epithelial integrity using stem cells, the clinical trial NCT04626583 is a Phase I dose-escalation evaluation of subconjunctival administration of allogeneic bone marrow-derived MSCs in patients with persistent, non-healing corneal epithelial defects. The primary focus is on safety and tolerability. However, because results have not been posted yet, no conclusions regarding efficacy or healing outcomes can be drawn from the registry entry alone.

Building upon the concept of regenerative therapies for DED, recent clinical efforts have also begun to explore the therapeutic potential of cell-free approaches, particularly exosome-based eye drops derived from various stem cell sources. The clinical trials NCT05738629 and NCT06543667 are early-phase, open-label studies investigating the use of topically administered exosomes derived from stem cells as a novel therapeutic approach for DED. NCT05738629 is a Phase 1/2 single-arm study evaluating eye drops formulated with PSCs-derived mesenchymal stem cell exosomes (PSC-MSC-Exos) in patients with DED following refractive surgery or associated with blepharospasm, conditions marked by tear film instability and ocular surface inflammation. Twelve participants received topical PSC-MSC-Exo drops over 12 weeks to assess safety and symptomatic improvement. Although results have not yet been posted, the treatment aims to leverage the bioactivity of exosomes—containing proteins, miRNAs, and immunoregulatory molecules—to restore ocular surface homeostasis. Complementing this, NCT06543667 is a Phase 1 exploratory trial evaluating the safety and preliminary efficacy of limbal stem cell-derived exosomes (LSC-Exo) in patients with moderate to severe DED refractory to standard therapies. Following a 14-day standardization period with artificial tears, participants receive LSC-Exo drops (10 μg, four times daily) for 30 days, with outcomes tracked over a 12-week follow-up. Together, these trials reflect growing interest in exosome-based therapies as cell-free, regenerative treatments capable of modulating inflammation and enhancing epithelial healing in DED [147].

In summary, the expanding field of regenerative ophthalmology continues to demonstrate the therapeutic potential of stem cell-based and cell-derived interventions for a wide range of corneal diseases, from LSCD and keratoconus to DED and persistent epithelial defects. Autologous tissue-engineered products such as Holoclar^®^ and Nepic^®^ have paved the way for clinical translation, while investigational approaches, including mesenchymal stem cell therapies, limbal stromal transplants, and secretome-based eye drops, offer new hope for conditions with limited treatment options. Despite promising safety profiles and encouraging preliminary efficacy, challenges related to regulatory approval, product preservation, and manufacturing standardization remain critical barriers to widespread clinical adoption. Continued clinical trials and long-term follow-up will be essential to validate these therapies and integrate them effectively into routine ophthalmic care. Table 4 summarizes the clinical trials.

### 7.2. Applications for Glaucoma and Optic Neuropathy

Glaucoma is a neurodegenerative optic neuropathy, which is a group of conditions characterized by progressive loss of RGCs, often leading to irreversible blindness [148]. This condition is one of the leading causes of irreversible vision loss worldwide, affecting millions of people, with estimates projecting a rise to 111.8 million by 2040 [3]. Current glaucoma therapies primarily focus on reducing IOP through daily eye drops, laser, or surgery, marking a shift toward regeneration, neuroprotection, and precision drug delivery [149]. Modern directions in the treatment of glaucoma, providing a transition to regeneration, neuroprotection, and precise drug delivery, are associated with the use of stem cells, implantable devices, and gene therapy. Currently, several clinical trials are being conducted to evaluate the effectiveness of stem cell treatment (NCT01920867, NCT03011541) of various optic neuropathies, including glaucoma [150]. In some cases, functional improvements in vision, such as improvement in Snellen visual acuity (e.g., from 20/400 to 20/100), have been observed in patients with glaucoma without any serious adverse effects. These improvements are believed to stem from neurotrophic support and paracrine modulation of retinal damage. While various publications from the SCOTS Ι trial reported meaningful visual improvements and safety across different optic neuropathies, it is worth noting that specific outcomes for glaucoma-induced optic nerve atrophy were not detailed in all published SCOTS reports [150]. However, the efficacy of cell therapy for glaucoma is of concern—in phase I NCT02330978, it was shown that the injection of MSCs from one patient was tolerated without functional changes, while another patient experienced retinal detachment and no improvement in vision [148]. Another promising approach for continuous intraocular delivery involves the NT-501, an ECT implant meticulously engineered for the sustained and continuous delivery of CNTF directly to the retina [95] for therapy of primary open-angle glaucoma (POAG) [151]. This compact device contains approximately 200,000 genetically modified hRPECs that secrete human CNTF [152].

As of current findings, phase I clinical trials have established the NT-501 CNTF implant as safe and well-tolerated in eyes affected by POAG, with no serious adverse events attributed to the device itself or the active therapeutic agent. Furthermore, the implanted eyes demonstrated encouraging structural and functional improvements, indicative of biological activity. Specifically, visual acuity and contrast sensitivity exhibited less decline in study eyes compared with control fellow eyes, and median Humphrey visual field index and mean deviation measurements improved in study eyes, while showing a decrease in fellow eyes [153]. Crucially, implanted eyes displayed a significant increase in retinal nerve fiber layer thickness, as measured by both OCT and GDx VCC. This structural change was generally detectable within one to three months post-implantation and sustained throughout the 18-month study period. Based on these preliminary findings, a randomized, sham-controlled, masked Phase II clinical trial in patients with glaucoma is currently underway, including an extension phase evaluating the effects of dual NT-501 implantation (NCT02862938, NCT04577300).

Apart from BMMSCs, adipose-derived MSCs have also been explored to combat glaucomatous neuropathies. The Phase I/II single-arm study NCT02144103 aimed to evaluate the safety and preliminary efficacy of autologous adipose-derived regenerative cells (ADRC) in patients with open-angle glaucoma. However, the study lacked a control group, and to date, no outcomes have been published regarding changes in visual field, intraocular pressure, or neuroprotective effects. Overall, while each of these studies represents a promising and innovative approach to addressing glaucomatous neurodegeneration or optic nerve damage, most of the clinical trials, such as NCT02638714 and NCT03173638, have not published results to date. The absence of clinical data limits the field’s ability to assess the actual value and impact of these experimental treatments.

Modern glaucoma therapies focus on regeneration, neuroprotection, and precision drug delivery, using both stem cells and implantable devices. Stem cell treatments have yielded mixed results: functional vision improvements have occurred (e.g., 20/400 to 20/100), but efficacy concerns remain, exemplified by one patient experiencing retinal detachment and no visual improvement in a Phase I trial. Furthermore, the lack of published data limits the assessment of many cell therapies. Conversely, Phase I trials established the NT-501 ECT implant, which continuously delivers CNTF for Primary Open-Angle Glaucoma (POAG), as safe and well-tolerated. Implanted eyes showed encouraging biological activity: functional decline (visual acuity and contrast sensitivity) was reduced, and critically, a significant and sustained increase in retinal nerve fiber layer thickness was observed for 18 months. These positive outcomes have prompted ongoing randomized Phase II clinical trials.

### 7.3. Clinical Applications for Retinal Diseases

#### 7.3.1. Cell Therapy of AMD

For AMD, a leading cause of vision loss, cell replacement therapies primarily target the RPE cells, whose dysfunction and loss are central to the disease’s progression [154]. In dry AMD, several hPSC-derived RPE products have been tested. Early studies involved the subretinal injection of cryopreserved hESC-derived RPE cell suspension (MA09-hRPE) in patients with dry AMD (NCT01344993). Slight vision improvements were noted (0–15 letters), but the reliability of visual acuity measurements in advanced disease patients was cautioned [73,134]. Similarly, another trial of MA09-hRPE (NCT01674829) observed 1–9 letter improvements in dry AMD patients, though interpretation was unreliable due to improvements in untreated eyes as well [155]. Despite these, the OpRegen product from hESC-derived RPE cells (NCT02286089) showed stable or improved visual acuity (+2 to +24 letters) in patients with less severe vision impairment. A phase II trial for OpRegen was initiated in 2023 (NCT05626114) and is currently recruiting [132]. Furthermore, in trial NCT02590692, hESC-derived RPE was tested as a non-cryopreserved cell sheet on a synthetic membrane for dry AMD (CPCB-RPE1), with 4 out of 16 patients showing a visual acuity gain of >5 letters at 12 months, and allogeneic RPE cells surviving without clinically detectable intraocular inflammation or serologic immune responses [132]. Moreover, apart from hESC, hiPSCs have also been used to treat dry AMD. An autologous hiPSC-RPE product utilizing a biodegradable scaffold, poly lactic-co-glycolic acid, was applied for dry AMD in trial NCT04339764, becoming the second autologous hiPSC trial worldwide. However, no results have been published yet. Another approach for dry AMD is CNTO 2476, also known as palucorcel, a novel investigational cell-based product composed of human umbilical tissue-derived cells (hUTCs). In clinical trial NCT01226628, CNTO was administered subretinally in 32 patients with GA. The implant was well tolerated, but no significant clinical improvement was noticed [156,157].

For wet AMD, which involves uncontrolled vascularization, hPSC-derived RPE cell suspensions and cell sheets are also in clinical trials [158]. The first trial using an hiPSC-derived product for wet AMD was initiated in Japan (UMIN000011929), delivering a non-cryopreserved, autologous hiPSC-derived RPE cell sheet (1.3 × 3 mm) without immunosuppression [69]. Graft survival was observed at 4 years, and visual acuity remained stable without additional anti-VEGF treatment [69,159]. In 2015, an hESC-derived RPE monolayer on a polyester membrane (PF-05206388, NCT01691261) was transplanted into two patients, leading to improved visual acuity with >20-letter gains at 12 months, marking clear signs of efficacy [160,161]. While this improvement decreased over 5 years, graft persistence was suggested by continued pigmentation. A non-cryopreserved RPE suspension product (hESC-RPE, NCT02749734) for wet AMD was also used, showing 11–26 letter improvements in visual acuity in all three patients [162,163]. A trial (UMIN000026003) initiated delivering cryopreserved HLA-haplotyped allogeneic iPSC-derived RPE cell suspensions without immunosuppression, resulting in stable or improved vision in five patients, with graft retention confirmed at one year [158]. It is worth noting that most early clinical trials involving the use of cells to treat AMD have not shown high efficacy, highlighting the difficulty of achieving stable therapeutic levels and translating results in vivo and the need for further optimization of delivery platforms. These findings underscore the challenge of achieving consistent therapeutic levels in vivo and highlight the need for further optimization of encapsulated delivery platforms [164].

Mechanistically, durable benefit from RPE therapy requires a polarized, adherent monolayer on Bruch’s membrane that resumes outer-segment phagocytosis and barrier/transport functions. Suspensions can boost local trophic tone but often struggle to re-form a stable sheet, whereas scaffolded monolayers/strips aim to restore apical microvilli–photoreceptor apposition and MerTK-dependent phagocytic flux, thereby stabilizing photoreceptor metabolism and the outer blood–retina barrier [47,59].

Although ocular sites can attenuate alloimmunity, clinical trials have shown immune engagement against RPE grafts. Reports include clinical rejection signals (e.g., progressive subretinal changes consistent with slow rejection at ~12 months in an RPE patch recipient [75]) and immune activation detected in peripheral assays in some recipients after RPE transplantation [76]. These observations support the use of peri-operative immunosuppression and the exploration of HLA-aware approaches to mitigate rejection risk in AMD.

Overall, cell replacement therapies for Age-related Macular Degeneration (AMD) target RPE cells. Results for dry AMD are generally slight or mixed: some studies showed minor visual improvements (e.g., 0–15 letters), although the OpRegen product demonstrated better stability or visual acuity improvement (+2 to +24 letters). In contrast, wet AMD trials have demonstrated clearer efficacy, including an RPE monolayer causing significant visual acuity gains of >20 letters at 12 months in two patients, and other suspension products showing 11–26 letter improvements. Despite these successes, the majority of early clinical trials overall lack consistently high efficacy, necessitating the ongoing optimization of delivery methods.

#### 7.3.2. Cell Therapy of Retinitis Pigmentosa

Cell-based therapies for RP focus on either replacing lost or dysfunctional cells or delivering trophic factors that promote the survival and function of remaining retinal cells [22]. Several types of stem cells are being investigated in clinical trials for RP. One of them is RPCs, which can differentiate along the retinal lineage. RPCs have been tested in NCT02320812 and NCT03073733. They showed low visual acuity improvements but a good safety profile, peak contrast sensitivity, and visual ability with hRPC injections [165]. While RPCs are generally free of ethical concerns and biosafety issues compared with other stem cells, directing their precise differentiation remains a challenge [166]. Human embryonic stem cell-derived RPE (hESC-RPE) has also been explored. In NCT03963154, allogeneic hESC-RPE cell sheets delivered in gelatin achieved stabilization of nystagmus and fixation [167]. But it should be noted that allogeneic hESC-derived products are subject to immune rejection, necessitating immunosuppression, though some studies suggest low immunogenicity [168]. Another approach for therapy of RP is iPSC-based therapies, including retinal organoids, which are also particularly promising as they can be derived from patient-specific cells or HLA-matched donors, potentially circumventing immune rejection and ethical concerns associated with ESCs [169]. For example, the transplantation of allogeneic hiPSC-derived retinal organoid sheets into RP patients has shown significant advancements (jRCTa050200027), reporting safety and stable survival of the organoids for up to two years, with slowed disease progression, though without significant visual changes [170].

Other kinds of cells, such as MSCs, are being investigated for their trophic support capabilities in RP [171]. Clinical trials such as NCT01068561 and NCT01560715 have evaluated autologous MSCs, reporting initial safety and transient improvements in vision-related quality of life, but with effects often not sustained long-term [172,173]. While MSCs offer advantages like low immunogenicity and ease of collection, their primary role is neuroprotective rather than cell replacement, and some trials have noted complications like fibrous tissue proliferation or retinal detachment [174,175]. Ongoing trials like NCT05413148 and NCT05786287 are further exploring the efficacy of MSCs for RP. Cell-based therapies for RP show encouraging safety across modalities—RPCs, hESC-RPE, iPSC-derived retinal organoids, and MSCs—with hints of functional benefit but limited, often transient, gains in visual acuity. Key hurdles remain—directing precise differentiation and durable integration for true cell replacement, managing immune risk for allogeneic products, and the mainly neuroprotective (not restorative) action of MSCs with occasional procedure-related complications. Ongoing, longer, and better-standardized trials—potentially combining cells with trophic or gene therapies—are needed to translate these early signals into durable vision improvement.

For retinal progenitor/photoreceptor strategies, true vision restoration depends on synaptic wiring, not donor survival alone. Dissociated precursors often show limited structural incorporation and, in late-stage models, apparent functional gains can partly reflect material transfer rather than robust synaptogenesis; prioritizing ribbon-synapse assembly (ELFN1/LRIT3→mGluR6/TRPM1) and constraining material exchange are active levers to convert survival into circuit function [62,176,177].

In conclusion, cell therapies for RP, encompassing RPCs, hESC-RPE, iPSCs, and MSCs, consistently demonstrate encouraging safety, yet functional benefits remain limited and often transient. While RPCs showed good safety but low visual acuity gains, and iPSC-derived organoids demonstrated stable survival with slowed disease progression but without significant visual changes, neuroprotective MSCs offered only temporary improvements and carry risks like retinal detachment. Key challenges across all modalities include achieving precise cell differentiation, managing immune rejection for allogeneic products, and transitioning from primarily neuroprotective action to durable functional integration.

#### 7.3.3. Cell Therapy of MacTel

MacTel type 2, a neurodegenerative retinal disease, is now addressed by an approved cell-based gene therapy: Revakinagene taroretcel (revakinagene taroretcel-lwey; Encelto™) [96]. Approved on 5 March 2025, in the USA, it is the first FDA-approved treatment for this condition. ENCELTO™ is an encapsulated cell-based gene therapy containing 200,000–440,000 allogeneic RPE cells that express recombinant human rhCNTF, delivered as a single-dose intravitreal implant. The rhCNTF is believed to promote photoreceptor survival by initially targeting Müller glia and inducing a signaling cascade. Clinical studies have shown sustained release of CNTF for up to 14.5 years [178]. In phase III NTMT-03-A (NCT03316300) and NTMT-03B (NCT03319849) studies, the revakinagene taroretcel implant significantly delayed photoreceptor degeneration, indicated by a significant reduction in the rate of change from baseline in ellipsoid zone loss over 24 months compared with a sham procedure [96]. Specifically, ellipsoid zone loss was reduced by 56.4% in NTMT-03-A and 29.2% in NTMT-03-B. In the phase II NTMT-02 study (NCT01949324), the implant also slowed retinal degeneration progression [179]. The most frequently reported adverse reactions included conjunctival hemorrhage, delayed dark adaptation, foreign body sensation, eye pain, and suture-related complications. Serious adverse reactions were rare. The implant was well tolerated across various patient populations. Overall, ENCELTO™ offers a disease-modifying benefit that stabilizes retinal structure rather than restoring vision, setting a new standard and platform for earlier or combination approaches.

#### 7.3.4. Cell Therapy of Stargardt

For Stargardt’s disease (STGD1), a common form of juvenile MD, clinical trials have evaluated the safety and effectiveness of subretinal injection of hESC-RPE cell suspension [180,181]. Studies like those involving MA09-hRPE (NCT01345006, NCT01469832, NCT01625559) showed mixed long-term visual function outcomes [155,180,182]. While no uncontrolled growth or inflammatory responses were found, microperimetry indicated no benefits at 12 months in some cases, and careful implementation of protective treatments was suggested due to the slow progression of the disease [182]. Also, MSCs have been utilized, with a phase II study involving suprachoroidal implantation of adipose tissue-derived MSCs in four patients showing improvements in visual field and acuity without systemic or ocular complications. However, the small sample size limited conclusive statistical analysis [182].

#### 7.3.5. Cell Therapy of DR

In DR, a vascular complication of diabetes, pericyte loss and inflammation contribute to retinal damage [183,184]. Conventional therapies have limited benefits, as pericyte loss is irreversible, and MSC-based treatments show promise in the therapy of DR [183,185]. For example, it was shown to result in a reduction in macular thickness and a significant improvement in BCVA in patients with non-proliferative DR, suggesting it as a safe and efficient therapeutic option, especially during the non-proliferative stage [183]. Furthermore, MSC-derived EVs have been investigated for their potential to alleviate inflammation and promote retinal vascular repair. These exosomes are also reported to prevent cell senescence and protect the outer blood-retinal barrier [100]. While the therapeutic potential of hPSC-derived products is evident, especially with emerging efficacy data for AMD, DR, RP, and Stargardt’s disease, challenges persist. Across AMD, RP, MacTel, Stargardt, and DR, completed and going clinical trials of cell therapies (Table 5) show a solid safety profile and emerging disease-modifying signals—most notably the FDA approval of ENCELTO™ for MacTel—alongside structural/functional stabilization in select RPE- and MSC-based trials. However, durable vision restoration remains limited; progress will hinge on precise photoreceptor/RPE integration, immune and delivery optimization, standardized endpoints, and earlier or combination (gene/trophic) approaches.

Beyond academic interest, the commercial dimension of cell therapies for retinal diseases is also of particular importance as several companies have already embarked on clinical programs. For example, Lineage Cell Therapeutics is developing the OpRegen program (RPE cells derived from embryonic stem cells) for dry age-related macular degeneration, which has completed phase 1/2a studies with encouraging safety and efficacy data. At the same time, jCyte has completed a phase 2b study for the administration of retinal progenitor cells to patients with retinitis pigmentosa, while Astellas has moved forward with early clinical trials of the MA09-RPE program in patients with macular degeneration and Stargardt disease. These examples demonstrate that the clinical translation of cell therapies has already entered the practical application phase, with clear prospects for future clinical exploitation.

#### 7.3.6. Stem Cell Therapy for Inherited Retinal Diseases

Τhe implementation of stem cell therapies, classified as advanced therapy medicinal products (ATMPs), holds enormous promise for pediatric patients suffering from inherited ocular disorders. These conditions, which include congenital anomalies and early-onset diseases, frequently subject children to a lifetime of severe vision impairment and/or blindness, accounting for up to 60% of blindness among infants and affecting an estimated 1.4 million visually impaired children under the age of 16 worldwide. The accessibility and immune-privileged nature of the eye facilitate the development of these novel therapeutic strategies [186,187]. Stem cell interventions are strategically aimed at inherited retinal diseases (IRDs) that are prevalent causes of blindness in early life, such as LCA, STGD, and RP [131,187]. LCA is recognized as a severe, early-onset retinal dystrophy that is believed to account for approximately one-fifth of cases of childhood blindness. STGD, an inherited form of juvenile macular degeneration, typically manifests symptoms starting in the later stages of infancy and extending into early adulthood [186].

These IRDs are characterized by the progressive degeneration and irreversible loss of specialized cell populations, PRs, and/or RPE cells [188]. As these cells are unable to regenerate naturally, stem cell therapy seeks to replace them by generating new retinal cells to substitute the injured photoreceptor cells and the outer nuclear layers [188]. Therapeutic strategies in development involve the generation of photoreceptors from both embryonic stem cells (ESCs) and induced pluripotent stem cells (iPSCs), which are expected to impact a wide range of inherited blindness conditions [186].

Recent quantitative systematic reviews suggest that stem cell transplantation is a potentially effective and safe therapeutic option for patients with RP or STGD. For STGD, improvements in best-corrected visual acuity (BCVA) have been reported, demonstrating statistically significant visual improvement at both 6 months (MD = −0.14 logMAR) and 12 months (MD = −0.17 logMAR) post-treatment. Conversely, while RP patients showed significant BCVA improvement at 6 months (MD = −0.12 logMAR), studies indicate that the long-term vision improvement may be limited for this condition [188]. Specific clinical trials targeting pediatric patients have been conducted in this area. For instance, one study involving patients with RP, whose median age was 13.4 (9.0–17.0) years, demonstrated that Umbilical Cord Mesenchymal Stem Cells (UCMSCs) injected into the suprachoroidal space showed the best BCVA improvement at 6 months post-treatment [188,189]. Transplanted stem cells achieve their beneficial effects through multiple mechanisms in the retina, including the secretion of neurotrophic factors, upregulating anti-apoptotic genes, providing immunomodulation and anti-inflammation, and functioning as cell replacement.

Cell replacement strategies are also applied to address corneal damage, particularly limbal stem-cell deficiency. This deficiency, often resulting from burn injuries to the eye, causes corneal destruction and can affect pediatric patients [186]. A somatic cell therapy has been developed where autologous limbal stem cells, explanted from a healthy eye, are subjected to ex vivo expansion culture to generate a curative patch. This technique has been evaluated in clinical trials that included some children (age range: 14–80, median: 46.5 ± 14.4 years). This approach requires only a small explant of healthy tissue to be cultured for 2–3 weeks before transplantation and has been cited as a cost-effective alternative compared with conventional corneal transplants [186].

The development and deployment of ATMPs for children introduce unique complexities. A critical challenge in treating rare genetic disorders is the youth of the pediatric patients. Logistical factors, such as the preference for the portability of the ATMP versus the transportation of the infant or juvenile patient to the manufacturing site, must be considered during clinical trial design. Ultimately, these cell and gene therapies offer the real prospect of curative treatment, which provides substantial quality of life benefits—such as the ability for children with disease to attend school and associate with peers—compared with requiring life-long medication [186].

#### 7.3.7. Stem Cell Therapy for Limbal Stem Cell Deficiency and Corneal Opacification

The field of ocular surface reconstruction for limbal stem cell deficiency (LSCD) has been significantly advanced by the introduction of Simple Limbal Epithelial Transplantation (SLET), a novel technique developed in 2012 [190,191,192,193]. LSCD, often caused by chemical or thermal injuries, leads to corneal conjunctivalization, vascularization, and eventual vision loss, potentially necessitating interventions like keratoplasty or keratoprosthesis [190,191,193]. Before SLET, primary surgical options included Conjunctival Limbal Autograft (CLAU) and Cultivated Limbal Epithelial Transplantation (CLET) [190]. CLAU required large biopsies (up to 120° of corneal circumference), risking iatrogenic LSCD in the healthy donor eye. CLET reduced donor tissue requirement but necessitated complex, specialized tissue culture expertise and expensive Good Manufacturing Practice (GMP) facilities for the ex vivo expansion of cells, along with requiring a two-stage surgical procedure [192].

SLET revolutionized autologous limbal stem cell transplantation by combining the benefits of CLAU and CLET while circumventing their inherent challenges. The procedure involves harvesting only a small piece of limbal tissue (e.g., a 2 × 2 mm biopsy, divided into 8–15 explant pieces) from the healthy eye [193]. These pieces are then directly transplanted onto the affected eye, following the surgical removal of abnormal tissue, in a single operation. The explants, acting as a “mini-limbus,” are fixed onto an amniotic membrane (AM) scaffold using fibrin glue, allowing the cells to expand in vivo and typically leading to complete reepithelialization within two weeks [191,192]. The efficacy of SLET is comparable to CLET for adults (anatomical success around 72.6% for SLET versus 70.4% for CLET). It demonstrates superior outcomes for pediatric patients (77.8% anatomical success for SLET versus 44.5% for CLET). This simplified and highly effective approach has contributed significantly to improving the treatment landscape for severe corneal surface diseases that might otherwise require more complex interventions or artificial implants, such as the Boston KPro [190,191].

The utility of SLET in Low and Middle-Income Countries (LMIC) is particularly pronounced due to its substantial economic and logistical advantages over cultivated techniques. The development of SLET, which originated in India, was explicitly analyzed from an Indian perspective. Crucially, SLET eliminates the need for expensive, specialized tissue culture facilities, highly skilled labor for cell expansion, and ongoing investment required to meet regulatory compliance for ex vivo expansion. The economic analysis estimates that SLET provides average cost savings of approximately US$6470.88 for adults and US$6673.10 for children compared with CLET, meaning the cost of SLET is roughly 10% of the cost of CLET for adults and 8% for children [191]. Furthermore, SLET requires only a single surgery, reducing demands on the healthcare system and the patient, and surgeons can be trained to perform the procedure in less than a week [191]. This financial accessibility and ease of replication make SLET a viable, cost-effective, and highly accessible treatment strategy in resource-constrained settings, expanding access to stem cell therapy for LSCD globally. The overall simplification offered by the SLET technique, which allows cells to slowly grow out of small tissue explants to form a new cornea, represents a financially attractive and much more accessible approach to treating vision loss [190,191].

In the context of severe corneal opacification, SLET also enhances the prospects of subsequent vision-restoring procedures, such as keratoplasty, which often serves as an alternative to keratoprosthesis. SLET successfully stabilizes and improves the corneal environment, sometimes promoting the self-clearing of the stroma. It is recommended that major procedures like penetrating keratoplasty (PK) be delayed for at least a year post-SLET, as performing PK simultaneously with SLET is correlated with a higher risk of failure [193]. Therefore, SLET acts as a necessary and highly effective biological reconstruction step, improving the environment for future surgical rehabilitation and providing a viable pathway toward biological corneal repair as an alternative to permanent artificial keratoprosthetic solutions. SLET has thus been widely adopted, largely replacing CLET as the preferred option for patients with LSCD in numerous hospitals internationally [190,193].

## 8. Safety and Adverse Events in Ocular Cell Therapy

The discussion regarding adverse events and safety issues in stem cell clinical trials focuses on risks related to both the surgical procedure and the intrinsic biology of the cells, such as oncogenesis and genetic mutations. Regarding complications related to surgical techniques and retinal detachment, the standard route of cell administration, subretinal injection, carries risks related to subfoveal retinal detachment (Table 6). Overall, clinical trials have reported adverse events such as the formation of epiretinal membranes (ERMs) and retinal detachment, which required treatment [174]. Even with careful vitrectomy and cell delivery, there is always a risk of donor cell leakage, proliferative vitreoretinopathy, and graft displacement. Sheet delivery is more complicated surgically, as it requires a much larger incision in the retina (retinotomy). In an RPE sheet trial, severe ERM formation was reported in one patient, while in a Japanese iPSC-RPE injection trial (UMIN000026003), all patients developed ERMs, which was considered to be related to the surgical procedure. A recent Japanese trial involving the implantation of organoid retinal sheets in patients with advanced retinitis pigmentosa (RP) reported that creating retinal detachment (to create space for the graft) in retinas with glaucoma was the most difficult part of the operation, although no serious adverse events such as detachments or hemorrhages were reported in this particular study [170].

The issue of oncogenesis and ectopic tissue proliferation is a central safety concern, particularly with Embryonic Stem Cells (ESCs) and induced pluripotent stem cells (iPSCs) due to their ability to form teratomas (tumors containing cells from all three germ layers). The basic strategy for resolving this issue is to ensure that the cells being transplanted are fully differentiated in vitro so that they are no longer proliferative. In addition, regulatory bodies place particular emphasis on confirming the absence of undifferentiated cells in populations intended for transplantation. When RPE cells are administered as a sheet, they are less likely to proliferate due to contact inhibition [155]. Unlike ESCs/iPSCs, Mesenchymal Stromal Cells (MSCs) do not appear to be inherently oncogenic, although extensive in vitro expansion carries the risk of accumulating genetic and epigenetic alterations, leading to senescence or potential transformation. In fact, aged MSCs can promote tumor growth in pre-existing tumors in the recipient through paracrine action [194].

To comprehensively address the problem of oncogenesis, it is recommended to limit in vitro expansion (usually to the fourth passage) to reduce the risk of genetic damage accumulation. Achieving genetic stability is a critical safety aspect, requiring rigorous analysis before clinical application. For this purpose, high-resolution methods such as Next Generation Sequencing (NGS) are used, which can detect very rare genetic alterations [195]. The issue of genetic mutations in Japanese iPSC trials was highlighted in the initial clinical study by the Mandai and Takahashi group (UMIM000011929), which used patient-specific iPSC-RPE sheets. This trial was terminated prematurely because the second patient developed oncogenic genetic mutations, which were likely due to the inherent genomic instability of iPSCs. As a result of this serious safety concern, the strategy shifted to the use of HLA-matched allogeneic iPSCs. This approach allows the creation of iPSC banks from healthy donors, which have been thoroughly tested for genomic stability (including Whole Genome Sequencing (WGS) and Whole Exome Sequencing (WES)) before clinical use, making them safer and more cost-effective compared with personalized autologous iPSCs [174]. In the recent trial with allogeneic retinal organoids (jRCTa050200027), safety was encouraging, as no unexpected overgrowth or tumor formation was observed in the grafts during the two-year follow-up [170].

The immunological recognition and clearance of allogeneic stem cell transplants within the subretinal space (SRS) remain significant challenges, despite the historical designation of the eye as an immune-privileged site, a status critically dependent upon maintaining the integrity of the Retinal Pigment Epithelium (RPE) monolayer [124,196,197]. Early graft rejection is predominantly characterized by innate immune activity, often circumventing the adaptive immune response, particularly within the first postoperative week [124]. This innate response involves immediate, rapid infiltration by macrophages (CD11b+, F4/80+) and neutrophils (Gr1, Ly-6G+), which actively infiltrate and engulf transplanted cells (phagocytosis/ADCP), leading to rapid graft clearance typically by postoperative day 7, often before significant T-lymphocyte infiltration (CD3-e) [124]. The local inflammatory response is initiated by cellular stress during graft preparation, resulting in the release of mediators such as the potent neutrophil chemoattractant KC/GRO/CINC (upregulated sixfold), along with increased levels of pro-inflammatory cytokines including IL-1b, IL-5, IL-6, and IL-12, driving inflammation and recruitment [124]. Furthermore, activated RPE cells and local glia function as regional antigen-presenting cells (APCs); resident phagocytic cells (microglia/macrophages) upregulate MHC Class II molecules to present extracellular antigens to CD4+ T-cells, while Müller glia upregulate MHC Class I to present intracellular antigens to CD8+ T-cells, processes often enhanced by increased expression of Type I Interferons and TNF-a signaling pathways [198]. Humoral immunity is also implicated, as host-acquired antibodies may cause donor cell death through Antibody-Dependent Cellular Cytotoxicity (ADCC) and Antibody-Dependent Cellular Phagocytosis (ADCP), regardless of the presence of the complement system in some instances [199]. Crucially, the outcome of the immune response is tissue-specific and context-dependent: while phagocytosis dominates rejection in wild-type mice, in models of retinal degeneration (rd1 mice), resident subretinal microglia adopt a non-phagocytic, non-inflammatory phenotype and actually support donor photoreceptor survival [200]. To overcome rejection, therapeutic strategies focus on minimizing surgical damage to the blood-retinal barrier [197], employing pharmacological agents such as minocycline or glucocorticoids to inhibit microglial/macrophage activation, utilizing methods like clodronate liposomes to deplete innate phagocytes, deplete innate phagocytes, and, when the barrier is breached, administering transient adaptive immunosuppression until local immune privilege is restored [124,197,199].

**Table 6:** Comparison of surgical routes for stem cell delivery in the eye (subretinal, intravitreal, and suprachoroidal).

Technique	Target/Delivery Method	Challenges/Risks	Impact on Therapeutic Outcome
Subretinal	Transplantation of hESC-derived RPE cells to the RPE and outer retina. Injection (e.g., 150 μL cell suspension) via cannula into the subretinal space [201]	-Highly invasive; requires pars plana vitrectomy (PPV). -Potential complications: vitreous loss, retinal detachment, retinal hemorrhage, retinal tear, atrophy. -Limited spread of injectate may restrict local efficacy [201]	-Enables localized therapy with potential photoreceptor rescue. -Subretinal space is immune-privileged, favoring graft survival. -Clinical studies reported BCVA improvement in multiple eyes and enhanced vision-related quality of life. -Transplantation in the “transition zone” optimizes integration between atrophic and healthy retina.
Intravitreal	Delivery of drugs, cells, or gene therapies directly into the vitreous cavity. Widely used for DME and neovascular AMD [201]	-Requires frequent injections due to the short half-life of agents. -Risk of endophthalmitis, retinal detachment, and vitreous hemorrhage. -MSC delivery may trigger pro-inflammatory effects and proliferative vitreoretinopathy (PVR) [201]	-Provides high bioavailability to retina/vitreous, bypassing some barriers. -Rapid onset of therapeutic action. -MSCs in glaucoma trials showed mixed results: generally, no functional improvement and rare severe complications (e.g., PVR) [148]
Suprachoroidal	Targeted delivery of drugs, genes, or cells (e.g., ADMSCs/UCMSCs) into the suprachoroidal space (between sclera and choroid) using microneedles (<1 mm) [202]	-Not immune-privileged; potential local inflammation with AAV vectors or cell therapy. -Technique optimization required (volume, viscosity, injection angle). -Mild to moderate AEs reported (e.g., pain, subconjunctival hemorrhage).	-Enables precise targeting of RPE, retina, and choroid, bypassing ILM and vitreous barriers. - Prolonged duration of action. -MSC/ADMSC implantation demonstrated improved visual acuity and visual field in degenerative diseases (AMD, Stargardt, RP). -Reduced anterior segment exposure lowers the risk of cataract and IOP elevation compared with IVT [201]

## 9. Future Directions of Cell Therapy in Ophthalmology

Cell-based regenerative strategies are rapidly advancing to address retinal degenerative diseases like AMD and RP. A major focus is on replacing the RPE, which is crucial for photoreceptor support. Clinical trials have explored transplanting RPE as cell suspensions versus preformed monolayer sheets on scaffolds [47]. Suspended RPE cells injected subretinally can improve neighboring retinal function but often struggle to re-form a lasting monolayer in vivo [47]. To enhance integration, several teams are implanting RPE monolayers on biomaterial scaffolds, essentially RPE “patches.” For example, hESCs-derived RPE on an ultra-thin parylene membrane showed safe integration and maintained function in an AMD trial [47]. Multiple Phase I/II trials are underway using scaffolded RPE grafts, including iPSC-RPE on human amniotic membrane and on a biodegradable PLGA scaffold [47]. These approaches aim to restore a polarized RPE layer in the diseased macula, with early results showing anatomical integration and hints of visual improvement. However, engineered RPE sheets entail more complex surgery than cell injections, and optimizing surgical devices and foldable biomaterials is an active area of research [47].

Single-cell suspensions (RPE or photoreceptors) are surgically simpler and can diffuse into micro-niches, yet they frequently show poor sheet re-formation (RPE) and low neuronal engraftment (photoreceptors); in advanced degeneration, benefits may include donor-to-host material transfer rather than robust synaptogenesis [47,176]. Biomaterial strategies (e.g., hydrogels) are being developed to reduce material transfer and improve the spatial placement of donor cells [177]. In contrast, organoid/retinal-sheet grafts deliver pre-patterned, layered tissue that preserves cell–cell architecture, improving survival, polarity, and host–graft synaptic connectivity in advanced models; genome-edited organoid sheets have restored fundamental light responses in blind retinas, and primate studies demonstrate anatomic integration in macular repair [83,203].

Beyond RPE, replacing lost photoreceptors and retinal neurons is a critical frontier for diseases such as RP. Early-stage trials are evaluating intravitreal injection of RPCs to regenerate photoreceptors. Notably, an allogeneic neonatal RPCs therapy (jCell) for RP has shown safety and signals of efficacy and is now moving into a pivotal Phase 3 trial in 2024. This therapy, delivered via minimally invasive injection, has received FDA regenerative medicine designation, reflecting its potential to broadly address hereditary retinal dystrophies regardless of the causative gene. Parallel efforts use photoreceptor precursors derived from stem cells: researchers have transplanted human stem-cell-derived photoreceptors (obtained from retinal organoids) into animal models. Historically, transplanting dissociated photoreceptors in late-stage degeneration models yielded modest vision gains, now understood to be partly due to material transfer—i.e., donor cells donating their proteins/organelles to host cells rather than fully engrafting [176]. Recent studies confirm that true integration of individual photoreceptors is rare (<5% of donor cells), and cross-species transplants do not readily exchange material [176]. Nonetheless, even without full synaptic integration, transplanted photoreceptors might rescue vision by delivering functional components to degenerating host cells [176]. Building on this concept, future cell therapies may combine cell grafts with methods to enhance connectivity or exploit trophic interactions. For instance, formulating grafts in supportive hydrogels can improve cell survival and placement [177]. Such biomaterial-assisted delivery could ensure transplanted photoreceptors remain in the target layer and form synapses rather than simply releasing vesicles.

Retinal organoid technology is further pushing the envelope of photoreceptor replacement. Retinal organoids (3D mini retinas derived from iPSCs or ESCs) can generate layered retinal tissue, including photoreceptors and RGCs. Transplanting sheets of organoid-derived retina is emerging to introduce new retinal tissue that can wire into the host. In a breakthrough study, researchers grafted retinal organoid sheets into end-stage retinal degeneration models and achieved host-graft synaptic connections. By CRISPR-editing the organoids to remove excess interneurons (knockout of Islet1), the grafts had a higher proportion of photoreceptors, which led to improved visual responses after transplantation [83]. Remarkably, RGCs in blind mice that received these genome-edited organoid sheets acquired light responses comparable to those of normal retina, responding to flicker and adapting to background light [83]. This indicates that transplanting a layer of lab-grown retina can, under the right conditions, restore fundamental vision signals—a clear validation of the organoid-based therapy approach [83]. Moving toward the clinic, organoid sheets are being tested in larger models. In 2024, a Japanese team implanted human ESC-derived retinal organoid sheets into the macular hole of macaque monkeys. The graft successfully filled the retinal defect, and the macular hole closed, with photoreceptors maturing in the graft, and some visual function improvement [203]. This work suggests that engineered retina tissue might be used not only for diffuse degeneration but even to repair focal retinal lesions (like macular holes) that are otherwise difficult to treat. Future directions will refine organoid derivation (e.g., enriching for specific retinal cells) and ensure grafts can reliably hook up with host circuitry. As regenerative ophthalmology progresses toward allogeneic cell products, researchers are leveraging gene-editing tools (like CRISPR-Cas9) to enhance cell therapy safety and efficacy. One key area is immunomodulation: modifying cells to evade immune rejection. Donor RPE or photoreceptor cells typically require immunosuppression when transplanted into patients, especially elderly AMD patients who cannot tolerate long-term immunosuppressive drugs [61]. To address this, scientists are creating “universal” cell grafts by knocking out major histocompatibility complex (MHC) molecules that trigger rejection. In 2024, a team demonstrated that macaque iPSC-derived RPE cells with CIITA gene knockout (abolishing MHC class II expression) had dramatically improved graft survival after transplantation into monkey eyes [61]. The MHC-II-deficient RPE provoked no inflammatory cell infiltration and successfully engrafted without immune attack [61]. Parallel efforts are exploring knockout of MHC class I or other immune checkpoints in human stem cell-derived RPE and photoreceptors, aiming for off-the-shelf cell therapy products that will not be rejected [61]. Another gene-editing application is autologous cell correction: a patient’s own cells can be reprogrammed into iPSCs, CRISPR-edited to fix disease-causing mutations, and differentiated into retina cells for transplantation. This personalized approach has been tested in laboratory models of inherited retinal diseases. For example, patient iPSCs with an RP mutation have been gene-corrected and turned into healthy photoreceptors in vitro [76]. While autologous iPSC therapy is time-consuming and costly, these advances suggest future cell therapies might combine gene therapy and cell therapy—correcting genetic defects ex vivo and delivering the repaired cells back to the eye.

The shift toward combined gene-and-cell approaches marks the evolution of regenerative medicine for the retina, as it seeks to integrate structural repair, functional optimization through genetic modification, and neuroprotection through long-term administration of agents. The use of genetically corrected grafts from iPSCs aims to achieve more effective functional integration of the transplanted cells into the host retina. Previous studies had shown that retinal organoid (RO) sheets used in clinical studies developed inner retinal cells, including bipolar cells (BCs). The bipolar cells within the grafts appear to competitively inhibit the formation of synapses between the host bipolar cells and the graft photoreceptors. To solve this problem, genetically engineered retinal organoids (gROs) were created in which the number of rod BCs or ON BCs was reduced after transplantation by deleting Islet1. In mice with retinal degeneration (rd1), transplantation of Islet1-deleted gRO sheets increased the number of photoreceptor synaptic connections per host BCs. This approach resulted in functional restoration, as retinal ganglion cells (RGCs) in these retinas (TP-rd1) gained photosensitivity comparable to wild-type retinas (WT). Furthermore, gROs restored fundamental physiological functions, eliciting ON, OFF, and ON-OFF responses to light stimuli, and exhibited adaptation to the photopic background. These results demonstrate that gROs can achieve simple light signal transmission and interact in a coordinated manner with the remaining neural network in advanced retinal degeneration [83]. In the context of gene therapy and correction, CRISPR/Cas9 technology has also been explored for in vivo restoration of RPGR expression (the gene responsible for X-linked retinitis pigmentosa, XLRP) in animal models to treat a severe form of inherited retinal degeneration [22,95,178].

In Phase I clinical trials in patients with primary open-angle glaucoma (POAG), the NT-501/CNTF implant was safe and well-tolerated. No serious ocular or extraocular adverse events attributable to the device or agent were reported. Importantly, implanted eyes showed structural and functional improvements, suggesting biological activity. Specifically, an increase in the mean retinal nerve fiber layer (RNFL) thickness of 10.16 μm was observed in the study eyes (versus 2.66 μm in the other eyes) using OCT, consistent with the effect of CNTF. CNTF delivery via ECT was stable and long-lasting, with high-dose implants maintaining the viability of encapsulated cells and secreting CNTF at effective levels in the human eye for at least 24 months (2 years) [95,153,178]). In addition, biomaterials act as the “plus” component for regulating cell behavior after transplantation. While hydrogels such as HAMC (hyaluronic acid and methylcellulose) have been shown to support donor cell survival, they have also been found to significantly reduce material transfer between photoreceptors, both in vitro (mitochondria) and in vivo (cytoplasmic GFP). This reduction was associated with reduced neurite outgrowth (such as PhNTs—photoreceptor nanotubes) and a transient delay in retinal reattachment after subretinal injection. Understanding how biomaterials can tune phenomena such as material transport—considered a potential therapeutic approach for protein augmentation—is vital for optimizing future gene-and-cell therapy strategies. Biomaterials are considered a “blank canvas” for future modifications to tune material transport for therapeutic use. Overall, the field is moving toward the development of multifunctional systems that combine the precision of genetic modification (such as CRISPR-correction) to enhance the function of cell grafts (gROs) with the effectiveness of long-term cellular delivery of biological agents (such as ECT/CNTF) [47,177].

Gene editing is also enabling novel cell products that secrete therapeutic factors. Instead of direct cell replacement, some ophthalmic therapies use living cells as “factories” to release neuroprotective molecules in the eye. A prominent example is encapsulated cell therapy for retinal diseases—Encelto (NT-501) for macular telangiectasia type 2. The approval of Encelto validates the concept of using a gene-edited cell line in an immune-isolating capsule to provide sustained retinal therapy. Importantly, because the cells are encased behind a semipermeable membrane, they evade immune rejection while still delivering drug-like benefits. Encapsulated cell therapy is now being explored in other conditions: for example, trials in geographic atrophy (advanced dry AMD) are testing whether CNTF delivery can protect photoreceptors and RPE from atrophy. There is even interest in applying CNTF implants or similar ex vivo gene therapies to glaucoma, to release neurotrophins that could protect optic nerve fibers. In the coming years, it is possible to see a pipeline of “cell pharmacies”—encapsulated RPE or other cell types engineered to secrete anti-VEGF, neurotrophins, or anti-inflammatory factors—entering clinical trials as adjunct treatments for chronic retinal diseases.

Regenerating the optic nerve presents unique challenges, as RGCs must not only survive injury (e.g., in glaucoma) but also regrow long axons from the eye to the brain.

The regeneration of the optic nerve does indeed pose unique challenges, as it requires both the survival of RGCs after damage (such as in glaucoma) and the regeneration of long axons from the eye to the brain. To support this scientific assumption, the study by Luo et al. provides documentation on an advanced injectable drug delivery strategy for the pharmacological treatment of glaucoma-related neurodegeneration [204]. The study developed a biodegradable and injectable thermoplastic, which, when loaded with pilocarpine and RGFP966 (an HDAC3 inhibitor), showed significant improvement in the attenuation of neurodegeneration through the suppression of oxidative stress, reduction in RGC loss, and enhancement of myelin growth and neuron regeneration. Specifically, HDAC3 inhibition is critical for regulating RGC atrophy and has been shown to promote neuron regeneration, which was confirmed by higher RGC density and restoration of optic nerve axon morphology in an experimental rabbit glaucoma model after treatment with the p-DMB + P + R system. These findings underscore that long-term dual drug delivery systems with multiple pharmacological actions (antioxidant, antiglaucoma, and neuroprotective) are promising approaches for the effective management of chronic neurodegeneration [204].

Future therapies are bifurcating into two strategies: neuroprotection (saving existing RGCs and fibers) and cell replacement (generating new RGCs and reconnecting the visual pathway). Cell-based neuroprotective therapies aim to slow or halt progressive RGC loss in glaucoma by creating a nurturing environment in the retina. For instance, intravitreal transplantation of MSCs has been shown to preserve host RGCs via paracrine release of growth factors. MSCs secrete factors like BDNF, CNTF, and GDNF that can enhance RGC survival under stress [205]. However, direct MSC injections carry risks (immune reactions, ectopic differentiation, or vascular obstruction), and clinical studies have had mixed results. An emerging alternative is to harness MSC-derived exosomes—nano-sized vesicles loaded with the MSC’s protective cargo. These exosomes can be injected into the vitreous to deliver trophic factors and microRNAs to retinal cells without introducing live cells [205]. Also, exosome therapy avoids many safety issues of cell transplants (no risk of uncontrolled cell proliferation or immune rejection) and can penetrate retinal tissues efficiently [205]. Thus, several groups foresee “cell-free” stem cell therapy using secreted vesicles to treat glaucoma. Early animal studies are promising, and translating this to human trials is a key near-term goal.

The more ambitious aim is to replace lost RGCs and enable new axons to reconnect the optic nerve. This is exceedingly complex—new neurons must integrate into the retina and grow through the optic nerve head to the brain—but recent advances offer hope. Scientists are using PSCs to derive retinal ganglion cell progenitors and testing their integration in retinal tissue. Under the NIH Audacious Goals Initiative, researchers have transplanted human iPSC-derived RGCs into retinal explants from primate eyes [206,207]. In ex vivo experiments, these donor RGCs can survive and exhibit neuronal activity, and multi-electrode recordings are probing whether they form synapses in the host retina [206,207]. While no human trial of RGC transplantation exists yet, these foundational studies will inform future cell therapy for optic neuropathies. Another parallel approach is in vivo reprogramming: rather than adding new cells, reprogram existing retinal cells to become RGCs or to regenerate axons. Pioneering work showed that gene therapy delivering Yamanaka factors to injured RGCs could restore youthful gene expression and induce some axon regrowth in mice [208]. By 2024, this “partial epigenetic reprogramming” approach has progressed to larger models, and a biotech company (Life Biosciences) announced plans for a first-in-human trial of an OSK gene therapy for optic nerve repair in glaucoma and optic nerve stroke. Though not a cell transplantation per se, such gene therapies essentially aim to convert Müller glia or surviving neurons into regeneration-competent cells, blurring the line between gene therapy and cell therapy.

The future of ophthalmic cell therapy is a multi-pronged effort. For retinal diseases, a pipeline from bench to bedside is seen—PSCs-derived RPE patches and photoreceptor cells are entering clinical testing, with translational studies bridging organoid science to surgical products [209]. Scalable manufacturing under GMP conditions has already enabled an iPSC-RPE cell therapy to reach IND approval in 2024 [209]. For optic nerve injury, groundbreaking research is tackling the long-distance regeneration problem with stem cells, engineered vesicles, and gene-edited rejuvenation therapies. Notably, cell therapies are disease-agnostic—a single cell product (like RPE or retinal progenitors) could potentially treat a range of conditions regardless of genetic cause. Over 100 human trials of PSC therapies are now registered worldwide [61], many in ophthalmology, underscoring the rapid maturation of this field. Challenges remain in ensuring safety (no tumorigenicity or ectopic growth), achieving functional connectivity, and navigating immune compatibility. Yet, each year brings tangible progress: from improved bioengineered retinal grafts that restore vision in animals [83] to real-world clinical milestones like the first FDA-approved cell-based gene therapy for MacTel type 2 retinal disease.

## 10. Challenges and Limitations of Cell Therapy

Stem cell therapy, despite its great promise in the treatment of ophthalmic diseases, faces a complex set of challenges and limitations that hinder its widespread clinical implementation. These challenges include safety issues, practical manufacturing limitations, and serious ethical dilemmas [210,211,212]. For example, the development of clinical cell therapies based on PSCs requires careful consideration of issues commonly associated with cell, tissue, and organ transplantation, and challenges remain in effective screening and immune matching, as well as meeting and enforcing existing regulatory requirements [213,214]. The most contentious barriers in stem cell therapy are the profound ethical and regulatory dilemmas, particularly concerning the use of ESCs [210,211,212]. While induced iPSCs offer a means to circumvent these embryo-related ethical concerns by reprogramming adult somatic cells, debates persist regarding the extent of gene expression changes introduced by the reprogramming process and the potential for these changes to elicit immune responses [215,216]. Also, there is a critical need for international agreement on quality control standards for deriving, characterizing, and monitoring cell lines to ensure their acceptability across various regulatory bodies, a challenge exacerbated by the long-term traceability required between donors and recipients, potentially for decades [217].

In addition to those, widespread clinical adoption of autologous therapies is limited by the challenges of manufacturing clinical-grade products that meet regulatory standards. For example, CLET, although supported by multiple trials, is not available in the United States because no current Good Manufacturing Practice (cGMP)-compliant processes have been developed for manufacturing this type of product, nor have any clinical trials evaluating this approach been approved by the FDA. For example, CLET products approved in the EU (Holoclar) and Japan (Nepic) use 3T3 feeder cells, serum, and antibiotics, methods that do not meet the FDA’s strict standards [71,140]. To address this obstacle, the standardized CALEC (Cultivated Autologous Limbal Epithelial Cell) standardized technique with a two-step construction was developed, designed to meet FDA requirements and using only FDA-compatible materials, without allogeneic or xenogeneic feeder cells. The development of regulatory-acceptable protocols requires the establishment of strict product release criteria. In general, it is necessary to establish standardized protocols for the isolation and ex vivo preparation of Mesenchymal Stem Cells (MSCs) before clinical application. Furthermore, to ensure reproducible clinical efficacy, the process of isolation, sorting, ex vivo expansion, purification, phenotyping, and follow-up testing must be fully documented. Furthermore, regulatory challenges extend to novel therapies, such as MSC-derived exosomes, where the absence of standardization of purification methods, separation, and profiling methods on a global scale may lead to controversial issues from different laboratory investigations. Furthermore, 3D bioprinting for personalized medical products does not fit into current regulatory categories, making it difficult to comply with global regulatory requirements for commercial purposes. This necessitates the need for a multidisciplinary global framework to resolve the legal issues associated with these advanced technologies [71,72,90,105]. The nature of autologous therapies, such as MSC transplantation, necessitates long-term surveillance, as the duration of the regulatory effects of MSCs remains unknown. Long-term monitoring is essential to ensure that MSC transplantation does not pose long-term risks to patients. In the CALEC Phase I/II study, the primary safety outcome was assessed at 18 months of follow-up, with participants completing follow-up by the 18-month visit. However, the results of the CALEC trial indicate the need for further studies with larger numbers of patients, at multiple centers, and longer follow-up to better define outcomes and factors predicting success. The high long-term success in the CALEC study precluded meaningful assessment of factors related to efficacy at the 12- and 18-month visits [71,90]. The production of active therapies is expensive, mainly due to their personalized nature and the need for specialized manufacturing processes. Although the manufacture of bioinks and 3D bioprinting are not expensive activities, the multidisciplinary nature of the overall process and the required supervision make it a high-cost process. This high cost raises ethical concerns, as the implementation of such technologies could lead to social stratification, resulting in them being accessible only to those with financial resources, while others would be unable to benefit from advanced therapies. It should be noted that, in contrast to the high costs associated with cell therapies, the use of alternative methods and materials can reduce expenses, as demonstrated by the example of Auro Kpro, a low-cost keratoprosthesis (estimated at $100 USD), compared with the Boston KPro ($5000 USD), which utilizes 3D printing technology [105,111].

Also, access to medical technologies and a comprehensive understanding of donors’ future use of their donated gametes, embryos, or somatic cells for iPSC generation remains crucial for protecting privacy and personal data [212]. Concerns also remain regarding potential tumorigenicity, particularly for cells such as ESCs and iPSCs [97]. While MSCs are generally considered safe with a low risk of tumor formation, rare instances of genetic aberrations in adult human MSCs in culture have been reported, albeit controversially [218]. For iPSCs, concerns about tumorigenicity were more prevalent, attributed to oncogenic factors used during induction, residual undifferentiated stem cells, and genetic aberrations from prolonged in vitro expansion [195]. Despite the availability of non-integrating vectors and stringent purification methods [219], some iPSC lines may still exhibit <99% identity to the starting material at the single-nucleotide polymorphism level and potentially contain missense mutations in critical genes, requiring rigorous quality control and further validation [217]. Ensuring the precise differentiation of stem cells into specialized ocular cell types, such as photoreceptors and RPE cells, and confirming their functional integration into existing neural networks remains a considerable technical hurdle [220]. Moreover, challenges persist in achieving scalability and consistency in producing therapeutic-grade cells, alongside difficulties in safe and efficient delivery to the delicate ocular environment, where conventional subretinal injections can lead to significant cell loss or injury post-transplantation [220]. The practical implementation of stem cell therapies in ophthalmology is also constrained by substantial economic, logistical, and operational limitations. The high cost and labor-intensive nature of producing autologous iPSCs for personalized medicine make it exceptionally expensive and not economically sound for widespread application [217]. Good Manufacturing Practice conditions, essential for producing therapeutic-grade stem cells, demand specialized facilities, intricate differentiation protocols, and stringent quality control, significantly driving up production costs and limiting global accessibility, particularly in low- and middle-income nations [16,221]. For instance, producing RPE cells from PSCs involves sophisticated bioreactors and labor-intensive procedures, which further restrict scalability and affordability [16]. Logistical challenges in therapy delivery extend beyond manufacturing expenses, compounding accessibility issues. Furthermore, the regulatory constraints and slow pace of clinical trials, particularly for advanced delivery techniques like 3D bioprinting, where legal regulations remain incomplete, delay commercialization and restrict access predominantly to wealthier regions [74]. The scarcity of suitable animal models that accurately mimic human disease pathophysiology, especially for complex conditions like glaucoma, also limits the relevant preclinical functional information available and underscores the reliance on transgenic mouse models for safety and effectiveness assessment [222].

Ophthalmic cell therapy products are manufactured under rigorous GMP conditions with extensive quality controls. For example, Holoclar^®^ (autologous limbal epithelial stem cells for LSCD) uses irradiated mouse 3T3-J2 feeder layers and defined growth media to expand a small (1–2 mm^2^) limbal biopsy. After primary culture, all cells are recovered and cryopreserved as an intermediate cell bank (ICB) [88]. On demand, thawed ICB cells are plated onto a fibrin matrix (with feeders) to form the final graft. This two-step (biopsy→ICB→graft) scheme provides manufacturing flexibility (Holoclar’s shelf-life is ~366 days) and allows batch testing before clinical use. By contrast, many MSC or iPSC-derived products are allogeneic and produced in larger batches. These typically use xeno-free, feeder-free culture systems (e.g., defined laminin or vitronectin substrates and humanized media) and cryopreserve final product aliquots or working banks for scalability. Throughout, strict GMP controls (media files, environmental monitoring) are enforced to ensure aseptic production. Quality control assays span identity, purity, differentiation potential, potency, genomic stability, and sterility. Identity is confirmed by marker expression: for instance, Holoclar cultures are characterized by corneal epithelial markers (cytokeratins CK3/12) and especially by the limbal “stem” marker ΔNp63α [88]. MSC products are tested by flow cytometry for the ISCT-defined phenotype (CD73+, CD90+, CD105+) [223], with depletion of hematopoietic markers (CD14, CD34, CD45, CD3)^−^ [223]. iPSC-derived RPE cells are similarly assayed for RPE-specific proteins (e.g., RPE65, BEST1, CRALBP, ZO-1) and lack pluripotency markers. Purity assays ensure absence of unwanted cells; e.g., MSC batches require ≥98% CD73/CD90/CD105 and ≤2% CD14/CD34/CD45 or CD3 [223]. Differentiation potential is routinely demonstrated for progenitor cells: MSCs must retain tri-lineage differentiation in vitro (osteogenic, adipogenic, chondrogenic), and iPSCs are checked for pluripotency (teratoma formation or tri-lineage marker expression) before differentiation to RPE. Potency assays are tailored to each therapy’s mechanism of action. For Holoclar, the key potency assay is the colony-forming (“holoclone”) assay based on ΔNp63α-bright cells: batches are screened by immunostaining to quantify “p63-bright” holoclones, and a minimum p63-bright percentage (derived from successful clinical batches) is required [88]. Indeed, a retrospective analysis of 91 patient grafts showed that higher percentages of p63-bright cells correlated significantly with clinical success (*p* ≈ 0.01) [88]. In practice, Holoclar cultures are only released if they meet the pre-defined holoclone specification [88]. For MSC-based ocular therapies, no single standardized potency assay exists; immunosuppression assays (e.g., mixed-lymphocyte reactions) are often used as surrogates. Regulatory guidance notes that a true in vivo mimicking potency test is an “unmet need,” and current practice relies on T-cell proliferation suppression or cytokine-release assays [223]. Emerging marker-based tests (e.g., IFNγ-induced IDO or PD-L1 expression) have been proposed but are still under validation [223]. For iPSC-derived RPE, potency is assessed by in vitro functional metrics: for example, differentiated RPE is tested for transepithelial electrical resistance and the ability to phagocytose photoreceptor outer segments (often compared with control RPE) before release. In all cases, karyotype/genomic stability is confirmed by G-banding or SNP/CNV arrays, and sterility is verified by pharmacopeial methods (culture and NAT assays for bacteria, fungi, mycoplasma, and endotoxin testing) [223]. Each batch must pass release criteria checked by a qualified person: for example, in a validated MSC process, the first release (after expansion) verifies sterility, mycoplasma, endotoxin, identity, purity, karyotype, and potency [223]. A second definitive release occurs post-cryopreservation, ensuring all tests (including viability and residual in vitro stability) meet specifications. Holoclar likewise requires sterility/mycoplasma certification and p63^bright^ potency before grafting [88]. These standardized manufacturing controls and potency tests underpin clinical translation and regulatory approval. The Holoclar example illustrates this: it was the first stem-cell ATMP approved in the EU (2015) precisely because its GMP processes and validated assays ensured consistent product quality [88]. With 72% of treated eyes achieving stable corneas at 1 year, efficacy was clearly linked to product attributes (i.e., sufficient stem-cell content [88]. In general, robust GMP production and quantitative release assays give regulators confidence in safety and potency, enabling licensure. They also allow comparison of outcomes to historical baselines or control arms and support reliable multi-center trials. As shown across various cell types, having defined identity/potency markers and scalable processes is critical for reproducibility and eventual commercialization of ocular cell therapies [223].

The burgeoning field of regenerative medicine offers profound therapeutic potential for currently intractable retinal degenerative diseases through the utilization of cell and stem cell-based therapies derived from hESCs and induced iPSCs. However, the global translation of these complex therapeutic approaches into widespread clinical use, particularly in Low and Middle-Income Countries (LMICs), is impeded by substantial barriers, demanding strategic solutions to ensure equitable access [211,224].

A primary impediment is the exorbitantly high cost of these Advanced Therapy Medicinal Products (ATMPs), which often makes universal distribution difficult and creates ethical concerns regarding distributive justice [211,225]. This lack of affordability is compounded by the reliance on out-of-pocket payments or external financing in many regions, such as Africa [226]. Infrastructural gaps present further critical obstacles, notably the lack of GMP-compliant facilities; adapting existing research settings to meet rigorous Good Manufacturing Practice (GMP) standards for aseptic processing is both costly and time-consuming [225]. Due to their living nature, cell therapies also require unique logistics features and maintenance of the cold chain to preserve viability. Cryopreservation, necessary for long-term storage, is costly, potentially unreliable, and induces significant stress on cells during freezing and thawing [225]. The complex process of transferring these living products further challenges timely patient delivery. Finally, regulatory readiness poses a formidable obstacle in LMICs, characterized by complex Clinical Trial Application (CTA) preparation, ambiguity, contradictory mechanisms, and often the absence of national guidelines for conducting clinical trials [225,226].

To enhance equitable access, key strategies focus on simplifying manufacturing and reducing costs. The development of off-the-shelf allogeneic products, derived from pluripotent stem cells (PSCs), is preferred as it facilitates mass production and standardization, significantly lowering the cost per dose compared with complex autologous methods [227]. Furthermore, establishing HLA-matched iPSC banks helps standardize production while potentially mitigating immunogenicity concerns [228]. Adopting low-cost and scalable manufacturing platforms, such as implementing automated, closed systems and leveraging Artificial Intelligence (AI) for real-time monitoring and quality control, is essential for achieving the economies of scale (scale-up or scale-out) necessary to reduce costs and increase product consistency and robustness [225,227]. Ultimately, overcoming these global barriers necessitates international collaboration among regulators, industry, and funding organizations, thereby establishing a supportive environment for advancing these groundbreaking therapies in resource-limited settings [225,226].

Ethical and regulatory considerations are central to the clinical translation of ophthalmic cell therapies and warrant a consolidated treatment to avoid repetition across disease-specific sections. Work with human embryonic stem cells (hESCs) raises long-standing questions about the moral status of embryos and the acceptable conditions under which embryos and oocytes are procured; contemporary oversight frameworks, therefore, require robust, documented informed consent that specifies scope of use, data sharing, storage, potential commercialization, and the absence of direct clinical benefit to donors, together with safeguards against undue inducement and minimization of medical risk to oocyte donors, as articulated in the ISSCR Guidelines for Stem-Cell Research and Clinical Translation from 2025 [229]. For induced pluripotent stem cell (iPSC)-derived and other donor-derived somatic tissue products, consent must additionally encompass broad future use of biospecimens and derived lines, genomic data sharing, and withdrawal limits, while compliance with regional data-protection regimes (e.g., GDPR in the European Union) is essential given re-identification risks inherent to sequence-level data. Clinical-grade manufacture further necessitates traceability and recall capabilities linking donor identity keys, cell banks, and released lots to downstream pharmacovigilance.

Cross-border regulatory divergences materially shape trial design and chemistry-manufacturing-controls (CMC) packages. In the European Union, cell and gene therapies are regulated as Advanced Therapy Medicinal Products (ATMPs) under Regulation (EC) No 1394/2007 [230] with centralized authorization by the EMA; the EPAR for Holoclar^®^—an autologous cultivated limbal epithelial product—illustrates how feeder-based epithelial expansion, intermediate cell banking, and post-authorization risk management can be justified within the ATMP framework. In the United States, products that exceed “minimal manipulation” or “homologous use” thresholds are regulated as biologics under the IND/BLA pathway within the HCT/P framework (21 CFR 1271; FDA overview), with programs such as RMAT designation providing expedited development for serious conditions. Japan’s PMDA/MHLW applies regenerative medicine-specific routes, including conditional/time-limited approval under the Act on the Safety of Regenerative Medicine and the Pharmaceuticals and Medical Devices Act, enabling earlier access with mandated post-marketing evidence. These jurisdictional differences translate into practical CMC choices—such as continued acceptance of 3T3-J2 feeder systems for some epithelial products in the EU/Japan versus a stronger expectation for xeno-free, feeder-free platforms in the U.S.—and into divergent expectations for immunogenicity, tumorigenicity, and long-term surveillance. Consolidating these ethical and regulatory elements in a single section allows subsequent mentions (e.g., feeder use for limbal expansion, genomic stability for pluripotent-derived RPE, data governance for iPSC biobanks) to be handled by cross-reference rather than repetition, while preserving product-specific details where directly relevant.

These collective practical limitations highlight the need for simplified regulatory frameworks, cost-effective technologies, and enhanced international collaboration to standardize procedures and increase access to stem cell treatments worldwide.

## 11. Conclusions

Modern cell-based therapies are reshaping the therapeutic landscape of ophthalmology, offering credible paths to tissue replacement (e.g., RPE/photoreceptor grafts), regeneration (LSCs for the cornea), and neuroprotection or vascular modulation in glaucoma and diabetic retinopathy. At present, these therapies remain investigational and are not standards of care; approvals are limited to specific products/indications and regions (e.g., Holoclar in the EU; Nepic/Ocural in Japan; Encelto for MacTel type 2 in the USA). Early clinical studies show encouraging safety and signals of activity, but conclusions are tempered by small cohorts, heterogeneous products, and short follow-up. For example, clinical evidence for cell-based therapies in ophthalmology is limited to small, early-phase (Phase I/II), open-label trials focused primarily on safety [174]. No large randomized controlled trials have been reported, and this important limitation in the evidence base must be explicitly acknowledged. In the absence of high-quality controlled data, conclusions about therapeutic efficacy remain provisional until confirmed by future randomized studies.

Also, the main hurdles remain consistent: reliable GMP manufacturing and differentiation, product characterization and potency, surgical delivery and long-term cell survival/integration, immunogenicity, and ethical–regulatory complexity. Moving from promise to practice will require rigorous, adequately powered trials with standardized endpoints, long-term surveillance, robust release criteria and biomarkers, scalable xeno-free manufacturing, and equitable access frameworks. If these gaps are addressed, cell-based interventions are poised to transition from experimental options to durable standards of care for major causes of vision loss.

Although non-cellular strategies—including gene therapy (e.g., voretigene neparvovec/Luxturna), pharmacological neuroprotection, retinal prostheses, and optogenetic approaches—represent important and complementary avenues, the present review intentionally focuses on cell-based therapies; readers are referred to recent comprehensive reviews of these non-cellular modalities for in-depth comparisons [231,232,233,234].

## Figures and Tables

**Table 1:** Comparison of stem cell types and their applications in ophthalmology and compares recent interventional clinical trials in 2023–2025.

Cell Type	Source	Differentiation Potential	Applications in Ophthalmology	Advantages	Limitations/Challenges	Development Stage
hESCs	Early-stage human embryos	High—can differentiate into RPE, neural, and corneal cells	AMD, Stargardt disease, RPE replacement	Pluripotent, well-studied protocols	Ethical concerns, immunogenicity	Clinical trials (I–II phase)
iPSCs	Reprogrammed somatic cells	High—similar to hESCs	RPE transplantation, genetic correction (e.g., Leber’s disease)	Autologous use is possible, avoiding ethical issues	Risk of mutations, complex reprogramming	Preclinical study
MSCs	Bone marrow, adipose tissue, and umbilical cord	Limited—mainly support or stromal roles	Neuroprotection, anti-inflammation, DR	Immunomodulatory, easy to isolate	Poor differentiation to RPE, short survival	Clinical trials (I phase)
LSCs	Corneal-conjunctival junction	Differentiate into the corneal epithelium	Corneal regeneration, LSCD	Autologous use, clinically established	Limited to LSCD, surgical collection	Approved (Holoclar^®^—EMA)
Genetically modified cells (GMCs)	Derived from iPSCs or other engineered cells	Varies depending on the source	Monogenic inherited retinal diseases	Personalized therapy potential	Safety, regulatory, and long-term expression control	Preclinical study
Comparative clinical trials: design, endpoints, and outcomes for 2023–2025						
Condition	Trial/Identifier	Cell type and delivery	Study design/N/Follow-up	Pre-specified endpoints	Key clinical outcomes (timepoint)	Notes
AMD (GA)	Stem cell-derived bioengineered RPE implant (CPCB-RPE1)—long-term follow-up) [79]	hESC-RPE on a synthetic scaffold; subretinal	Phase 1/2a; single-arm, open-label; *N* = 16 (15 implanted); median 3 y follow-up	Primary: Safety; Secondary: BCVA, multimodal imaging (OCT, fundus), IOP; systemic humoral immune monitoring	Safety met; implanted eyes more likely to gain >5 ETDRS letters and less likely to lose >5 vs. fellow eyes at median 3 years; implant stable in position; anticipated hemorrhage mitigated with surgical refinement (cohort 2) (years 3)	First peer-reviewed multi-year outcomes for scaffolded RPE in GA; efficacy signals vs. fellow eye; randomized data still pending [79]
AMD (GA)	OpRegen (RG6501)—sponsor/meeting 36-mo readout; Phase 2a ongoing (NCT05626114)	hESC-RPE suspension; subretinal	Phase 1/2a; single-arm, open-label; cohorts by GA severity; 36 months follow-up reported	Safety; BCVA; OCT (outer retinal structure, lesion coverage)	Mean +6.2 ETDRS letters at 36 months in less advanced cohort; greater lesion coverage associated with larger BCVA and structural gains; durability signals to 36 months (non-randomized)	Peer-reviewed primary paper pending for 36-months efficacy; Phase 2a GAlette enrolling (NCT05626114) [80,81]
AMD/RP (RPE loss)	Allogeneic iPSC-RPE strips (HLA-mismatched) [82]	iPSC-RPE strips (pre-formed); subretinal	First-in-human, Phase 1-type; single-arm, open-label; N = 3 (1 dry AMD, 2 MERTK-RP); ≥6–12 months follow-up	Primary: Safety/feasibility; Secondary: BCVA, OCT (strip position/continuity), AEs; systemic IS for 24 weeks	Acceptable safety with HLA-mismatch under short IS; anatomic graft survival on OCT; functional measures stabilized/improved in some eyes (early follow-up) [82]	Extends prior RPE scaffolds/suspensions with strip format; very small N; randomized data lacking
RP (advanced)	Genome-edited retinal organoid sheets—case series [83]	Patient-matched genome-edited retinal sheets; subretinal	Clinical case series; 2 eyes/2 patients; 24 months	Safety, anatomical survival; exploratory function	Stable survival of sheets and safety to 24 months; exploratory signals of local structural integration; functional endpoints limited in end-stage disease (24 months) [83]	Pioneering but very small; controlled functional efficacy not established
LSCD (cornea)	iPSC-derived corneal epithelial sheets [84]	iPSC-corneal epithelium sheets; ocular surface	Single-arm, open-label, first-in-human; N = 4; 52 weeks	Primary: Safety; Secondary: corneal epithelialization, BCVA, AS-OCT, neovascularization, QOL	Epithelialization restored and surface stabilized in most eyes; BCVA improved or stabilized by 52 weeks; no serious graft-related AEs; effect greater in less severe cases (52 weeks)	First iPSC-corneal sheet FIH study; small N; no comparator; durability >1 year needs further tracking
AMD (GA, earlier cohort)	Bioengineered RPE implant—early HLA-mismatch findings (safety/engraftment) [85]	hESC-RPE on scaffold; subretinal	Phase 1/2a subset; single-arm; HLA-mismatch	Safety/immune monitoring; imaging	No clinical signs of intraocular inflammation or serologic response despite HLA mismatch; RPE survival and functionality on imaging/histology (early)	Included here as mechanistic/immune context supporting the 2024 follow-up

**Table 2:** Comparative analysis with traditional transplantation.

Modality	Primary Indication(s)	Graft/Format	Mechanism of Benefit	Integration Requirement	Durability Considerations	Immunogenicity/IS	Surgical Complexity	Scalability
RPE sheet/patch (PSC-derived) [47,76]	AMD-GA/RPE loss	Polarized monolayer on a scaffold	Structural and metabolic support; OS phagocytosis; barrier/transport	High: adherent, continuous, polarized RPE on Bruch’s	Tied to monolayer integrity and immune milieu	Allogeneic risk; HLA-aware strategies emerging	Subretinal surgery/device handling	Batch manufacture; QA for polarity/purity
RPE suspension (PSC-derived) [47]	AMD	Single cells, subretinal	Trophic support ± local repopulation	Moderate: In vivo re-sheeting is inconsistent	Often early peaks; variable if the sheet does not reform	Similar to above	Subretinal injection	Easier to produce/freeze
Photoreceptor precursors/retinal organoid sheets [76,86]	RP/outer retinal degeneration	Suspension or laminated sheets	Neuronal replacement ± trophic aid	High for true vision restoration (synapses with bipolar cells)	Gains are short-lived if synaptogenesis is limited; sheets may be superior	Allogeneic risk	Subretinal surgery	Complex differentiation
MSCs (neuroprotection) [76]	RP, DR, glaucoma; ocular surface	Intravitreal/subretinal/subconjunctival	Paracrine immunomodulation and trophic effects	Low (no structural replacement)	Transient if cells do not persist; repeat dosing	Generally low; route-dependent safety	Injection-based	Readily scalable
LSC/epithelial constructs (Holoclar^®^, Nepic^®^, CALEC) [87,88,89,90]	LSCD	Autologous epithelial sheet	Structural resurfacing of the cornea	High: stable, avascular epithelium; limbal niche	Durable with niche/vascular control	Autologous minimal; allogeneic rejection risk	Ocular surface surgery	Autologous bespoke
KLAL/lr-CLAL (allogeneic limbal) [78]	Bilateral LSCD	Donor limbal tissue	Restores the stem cell pool	High	Variable; immune-mediated failure is common without IS	High; systemic IS typical	Ocular surface surgery	Donor dependent
Traditional corneal transplantation (PKP, DSAEK/DMEK) [91,92,93,94]	Corneal opacity/endothelial failure	Donor tissue	Tissue replacement	N/A	Good mid-term; endothelial attrition over the years	Rejection risk (esp. endothelium)	Microsurgery	Donor-limited

**Table 3:** Summary of pooled (or reported) BCVA findings from external publications.

**Disease or setting**	Retinal degenerations (mixed; includes AMD/RP/SMD across modalities)	Inherited retinal diseases (IRDs)	Optic neuropathies (context for neuro-retinal VA effects)	AMD (dry; selected early-phase cohorts)
**Cell modality (studies pooled)**	Mixed cell therapies (hPSC-RPE, MSCs, RPCs) [131]	Mixed stem-cell interventions [131]	MSCs (autologous/allogeneic) [7]	hESC-/iPSC-RPE (injection or patch) [7,132,133,134,135]
**Pooled/reported BCVA finding**	Overall modest BCVA improvement with substantial heterogeneity across designs and indications	Directionally favorable BCVA change, but wide CIs and non-uniform durability	Statistically significant BCVA gains in pooled analysis; clinical magnitude modest	Reported BCVA gains ranging from single-digit to ~+20 letters in small cohorts (varies by program); attenuation on longer follow-up in some series; no AMD-only meta-estimate in these sources
**Typical follow-up**	Mostly 3–12 months	Up to ~12 months	3–12 months	6–12+ months
**Notes/heterogeneity**	Authors emphasize BCVA limitations and recommend multi-metric outcomes; durability beyond 12 months is uncertain.	Recommends adding microperimetry/ERG; calls for standardized designs and longer follow-up.	Study quality and heterogeneity limit durability inference; included as supportive evidence in related neuro-retinal settings.	Outcomes depend on establishing a durable, polarized RPE monolayer; variability by format (suspension vs. patch) and baseline severity.

**Table 4:** Clinical trials of cellular therapies for corneal pathologies.

NCT Number	Study Name	Phases, Study Status	Conditions	Date of Start
NCT05279157	Autologous adipose-derived adult stem cell implantation for corneal diseases	II, Completed	Corneal diseases	19-04-2022
NCT04932629	To evaluate the clinical safety and efficacy of LSCs for the treatment of superficial corneal pathologies	I, Unknown	Corneal scars and opacities	07-2021
NCT04626583	Safety of locally delivered allogeneic AMSCs	I, Completed	Corneal defect	05-03-2021
NCT04615455	MSCs therapy of DED in patients with Sjögren’s syndrome	II, Completed	Keratoconjunctivitis Sicca, Sjögren’s Syndrome	03-11-2020
NCT04484402	Treatment of patients with inflammatory-dystrophic diseases of the cornea using autologous stem cells	I–II, Completed	Corneal ulcer, corneal disease, corneal dystrophy	03-10-2016
NCT03878628	Treatment with allogeneic adipose-derived MSCs in patients with aqueous-deficient DED	I, Completed	Dry eye, keratoconjunctivitis sicca, aqueous tear deficiency	16-10-2019
NCT03302273	Corneal epithelial stem cells and DED	NA, Completed	Dry eye syndromes, dry eye, ocular inflammation, ocular surface disease, ocular discomfort, blepharitis	01-02-2019
NCT02592330	LSCD treatment with cultivated stem cell (CALEC) graft	I–II, Completed	LSCD	01-08-2016
NCT02577861	Efficacy and safety of autologous cultivated LSCs transplantation (ACLSCT) for restoration of corneal epithelium in patients with LSCD	IV, Completed	LSCD	10-2015
NCT01562002	Safety study of stem cell transplant to treat limbus insufficiency syndrome	I–II, Completed	Limbus cornea insufficiency syndrome	03-2012

**Table 5:** Clinical trials of cellular therapies in retinal pathologies that are registered at the National Institutes of Health.

NCT Number	Study Name	Phases, Study Status	Results	Conditions	Date of Start
NCT06557460	A phase IIb clinical trial to assess the safety and efficacy of subretinal implantation of the CPCB-RPE1 implant in subjects with advanced dry AMD	II, not yet recruiting	no	Dry AMD, GA	10-2024
NCT06394232	Safety and efficacy of EYECYTE-RPE™ in patients with GA secondary to dry AMD	I–II, recruiting	no	RD, AMD, GA	04-06-2024
NCT05626114	A study to optimize subretinal surgical delivery and to evaluate the safety and activity of Opregen in participants with GA	II, recruiting	no	GA	23-03-2023
NCT05445063	Safety and efficacy of autologous transplantation of iPSC-RPE in the treatment of MD	I, recruiting	no	MD	08-2022
NCT04339764	Autologous transplantation of iPSCs-derived RPE for GA associated with AMD	I–II, recruiting	no	Dry AMD, GA	23-09-2020
NCT03944239	Safety and efficacy of subretinal transplantation of clinical hESCs derived RPE in the treatment of RP	I, unknown	no	RP	05-2020
NCT03305029	The safety and tolerability of sub-retinal transplantation of SCNT-hES-RPE cells in patients with advanced dry AMD	I, unknown	no	Dry AMD	05-2016
NCT03178149	A study of the safety and tolerability of ASP7317 in senior adults who are losing their clear, sharp central vision due to GA secondary to dry AMD	I, recruiting	no	GA, AMD	13-07-2018
NCT03046407	Treatment of dry AMD with RPE derived from hESCs	I–II, unknown	no	Dry AMD	06-09-2017
NCT02903576	Stem cell therapy for outer retinal degenerations	I–II, completed	no	AMD, Stargardt’s disease	08-2015
NCT02755428	Subretinal Transplantation of RPE in the treatment of AMD	I–II, unknown	no	Dry AMD MD	01-2018
NCT02749734	Clinical study of subretinal transplantation of hESCs derived RPE in the treatment of MD diseases	I–II, unknown	no	MD, Stargardt’s macular dystrophy	05-2015
NCT02590692	Study of subretinal implantation of hESC RPE cells in advanced dry AMD	I–II, unknown	no	Dry MD, GA	16-02-2016
NCT02286089	Safety and efficacy study of OpRegen for treatment of advanced dry AMD	I–II, active	yes	Dry AMD	01-04-2015
NCT01691261	A study of the implantation of RPE in subjects with Acute wet AMD	I, completed	no	Wet AMD	14-10-2021
NCT01674829	A study to determine the safety and tolerability of MA09-hRPE cells in patients with advanced dry AMD	I–II, terminated	no	Dry AMD	09-2012
NCT01625559	Safety and tolerability of MA09-hRPE cells in patients with Stargardt’s macular dystrophy	III, completed	no	Stargardt’s macular dystrophy	09-2012
NCT01469832	Safety and tolerability of sub-retinal transplantation of hESC-RPE cells in patients with Stargardt’s macular dystrophy	I–II, completed	no	Stargardt’s macular dystrophy	13-12-2011
NCT01345006	Sub-retinal transplantation of hESC-derived RPE (MA09-hRPE) cells in patients with Stargardt’s macular dystrophy	I–II, completed	no	Stargardt’s macular dystrophy	16-06-2011
NCT01344993	Safety and tolerability of sub-retinal transplantation of hESC-derived RPE (MA09-hRPE) cells in patients with advanced dry AMD	I–II, completed	no	Dry AMD	09-06-2011
UMIN000011929	A study of transplantation of autologous iPSC-derived RPE cell sheet in subjects with exudative AMD	I–II, completed	yes	Wet AMD	2013
UMIN000026003	A Study of transplantation of allogenic iPSC-derived RPE cell suspension in subjects with neovascular AMD.	I, completed	-	Wet AMD	2017
jRCTa050210178	Clinical research of allogeneic iPSC-RPE cell strip transplantation for RPE impaired disease	I–II, active	-	RPE impaired disease	2022
jRCTa050200027	Safety Study of allogenic hiPSC-retinas in RP	I, completed	-	RP	2020

## Data Availability

No new data were created or analyzed in this study. Data sharing is not applicable to this article.

## Abbreviations

ADMSCs	Allogeneic adipose-derived mesenchymal stem cells
ADRCs	Adipose-derived regenerative cells
AMD	Age-related macular degeneration
AI	Artificial intelligence
BCVA	Best-corrected visual acuity
BMMNCs	Bone marrow mononuclear cells
CAD	Computer-aided design
cGVHD	Chronic graft-versus-host disease
CNTF	Ciliary neurotrophic factor
DED	Dry eye disease
DR	Diabetic retinopathy
ECT	Encapsulated cell technology
ESCs	Embryonic stem cells
EVs	Extracellular vesicles
GA	Geographic atrophy
hAECs	Human amniotic epithelial cells
hESC-RPE	Human embryonic stem cell-derived retinal pigment epithelium
hESCs	Human embryonic stem cells
hUTCs	Human umbilical tissue-derived cells
IRDs	Inherited retinal dystrophies
iPSCs	Induced pluripotent stem cells
LSCD	Limbal stem cell deficiency
LSCs	Limbal stem cells
MD	Macular degeneration
MSCs	Mesenchymal stem cells
POAG	Primary open-angle glaucoma
PSCs	Pluripotent stem cells
RGCs	Retinal ganglion cells
RP	Retinitis pigmentosa
RPE	Retinal pigment epithelium
RPCs	Retinal progenitor cells
SCT	Stem cell therapy
